# Atomically preserved MXene quantum dots as a redox-responsive nanoplatform for light-controlled bidirectional ROS engineering

**DOI:** 10.1016/j.mtbio.2026.102864

**Published:** 2026-01-28

**Authors:** Dejia Hu, Tianhao Xia, Danyang Xiao, Bufeng Liang, Yuyi Li, Jinkun Li, Zhongliao Zeng, Jianxiong Ma, Yan Li

**Affiliations:** a School of Material Science and Engineering, University of Science and Technology Beijing, Beijing, 100083, PR China; b The Second School of Clinical Medicine, Zhejiang Chinese Medical University, Hangzhou, 310053, PR China; c Department of Nephrology, the First Affiliated Hospital of Zhejiang Chinese Medical University (Zhejiang Provincial Hospital of Traditional Chinese Medicine), Hangzhou, 310006, PR China; d Zhejiang Key Laboratory of Research and Translation for Kidney Deficiency-Stasis-Turbidity Disease, Hangzhou, 310000, PR China

**Keywords:** MXene quantum dots, Microneedles, ROS generation, ROS scavenging, Redox microenvironment

## Abstract

MXene quantum dots (MQDs) combine the intrinsic reductive properties of MXenes with the photoactivity induced by quantum confinement, positioning them as promising agents for dynamic redox regulation in therapeutic applications. However, their translation into practice has been limited by persistent synthetic issues, including transition-metal leaching and oxidative degradation. To address these challenges, a sodium ascorbate-mediated coordination and reduction strategy was developed for hydrothermal synthesis of structurally intact Ti_2_C MQDs with improved crystallinity and high titanium retention. The resulting MQDs exhibit a unique extension of optical absorption into the visible range, which facilitates efficient ROS generation under visible-light irradiation and confers potent antibacterial properties against pathogenic bacteria. Concurrently, the MQDs demonstrate broad-spectrum ROS scavenging ability. At the cellular level, they effectively reduced oxidative stress and inflammation while promoting M2 macrophage polarization. Capitalizing on these dual redox activities and excellent biocompatibility, a collagen–alginate microneedle patch (MQDs@Col-SA MN) was designed to evaluate their therapeutic potential. In a diabetic wound model, this system achieved ∼80 % smaller wound area than untreated controls at Day 10, while also outperforming a positive control dressing. This study represents the first report of structurally preserved MQDs capable of adaptive redox regulation, underscoring their utility as a versatile platform for microenvironment modulation and regenerative medicine.

## Graphical abstract

**Figure:**
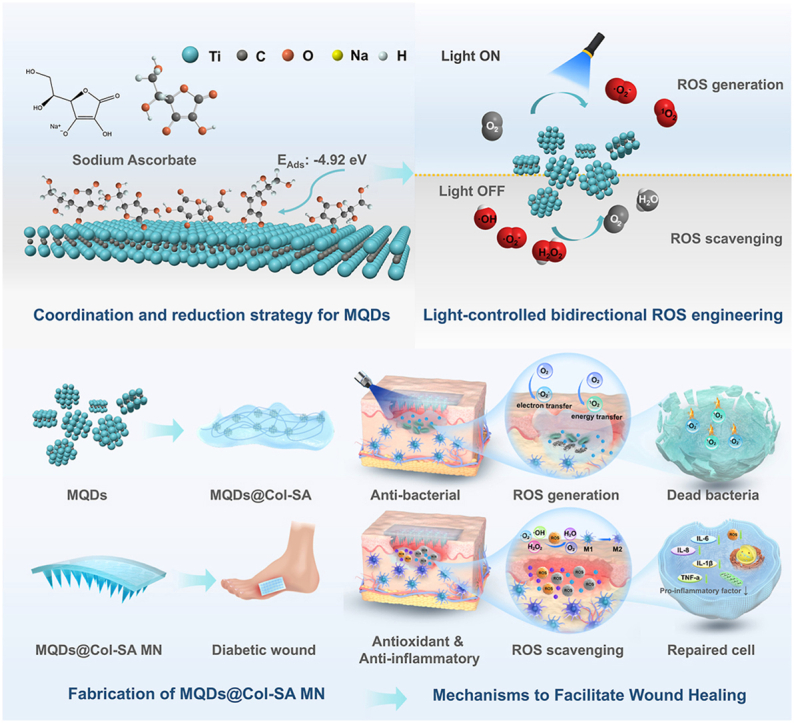

## 1 Introduction

Reactive oxygen species (ROS), including superoxide anion (· O_2_^−^), hydrogen peroxide (H_2_O_2_), and hydroxyl radicals (·OH), are chemically reactive molecules that play a dual role in biological systems. While short-term ROS bursts activate immune responses and cellular repair [1], persistent overproduction leads to oxidative stress and tissue dysfunction [2,3]. Thus, the precise modulation of ROS has emerged as a frontier challenge across materials science and biomedicine, underpinning applications ranging from antibacterial therapy and anti-inflammatory treatment to tumor inhibition and regenerative engineering [[4], [5], [6], [7]]. Central to these strategies is the development of smart redox-active nanomaterials capable of switching between ROS generation and scavenging in response to local microenvironments.

With advances in nanotechnology, diverse ROS-active nanomaterials, such as ZnO [8], MnO_2_ [9], MOFs [10], and carbon dots [11], have been explored. However, their redox behavior is primarily governed by microenvironmental conditions, and any observed switching results from passive environmental responses rather than active external control. To enable bidirectional ROS regulation, nanoscale heterostructures have been constructed through the physical assembly of discrete oxidative and reductive components [12,13]. However, these multicomponent systems are often plagued by internal electron transfer conflicts, kinetic mismatches, and a lack of integration, limiting their effectiveness in complex biological environments. This highlights the need for a monocomponent platform that intrinsically combines both oxidative and reductive capabilities [14], thereby eliminating interfacial barriers and enabling efficient, stable, and predictable ROS regulation.

In this context, MXene-derived quantum dots (MQDs) offer a compelling solution [15,16]. MQDs inherit the large specific surface area and inherent reductive properties of MXenes, enabling high reactivity toward ROS scavenging. Importantly, quantum confinement imparts MQDs with a size-dependent band gap that enables photoexcitation and subsequent redox transformations, providing the fundamental electronic basis for their light-activated ROS generation. Depending on external stimuli (such as pH, redox potential, and irradiation), MQDs can function as either pro-oxidants [16] or antioxidants [17], making them attractive for “all-in-one” ROS dynamic regulation. However, the synthesis of redox-efficient MQDs remains hindered by severe oxidative degradation during top-down fabrication. Excessive oxidation disrupts the crystal lattice and depletes transition metal elements [18,19], resulting in carbon-dominated byproducts with diminished activity [[20], [21], [22], [23]]. Although additive-assisted strategies have been attempted [24,25], they remain insufficient to stabilize edge structures and preserve transition metal sites, leaving the intrinsic instability of MQDs largely unresolved.

In this work, sodium ascorbate was introduced as a functional additive to stabilize MXene edge architectures and suppress excessive oxidation during quantum dot synthesis. This strategy preserved the layered structure and Ti sites, markedly improving crystallinity and redox efficiency. The MQDs exhibited reliable ROS scavenging under physiological conditions and effective ROS generation upon photoactivation. To translate this functionality into a practical therapeutic platform, MQDs were integrated into a collagen-sodium alginate microneedle patch (MQDs@Col-SA MN) for tissue penetration and controlled release. In diabetic wound models, this system modulated the inflammatory microenvironment, promoted tissue regeneration, and achieved superior healing outcomes compared to conventional treatments. Collectively, these findings establish atomically preserved MQDs as a robust redox nanoplatform that resolves long-standing synthetic challenges and demonstrates their successful translation into a microneedle-based system, offering spatiotemporal precision for phase-adaptive wound therapy and broader regenerative applications.

## 2 Results and discussion

### 2.1 Preparation and characterization of the MQDs

**Fig. 1:**
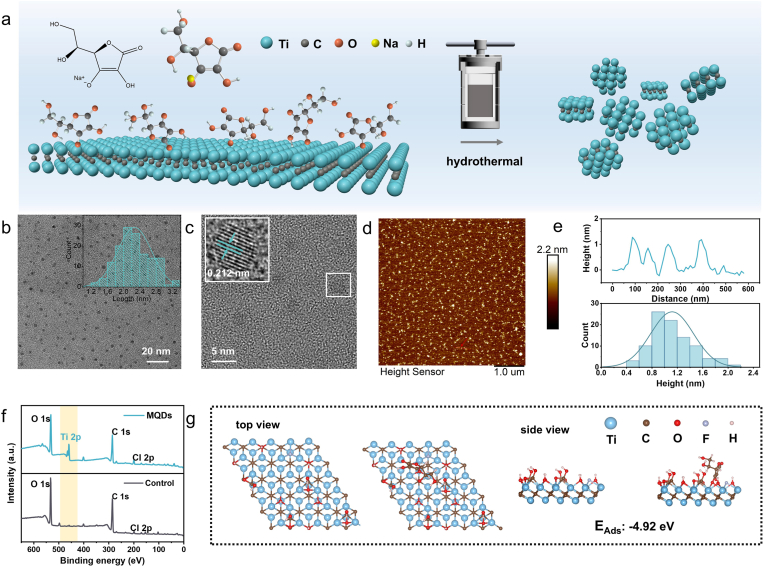
Characterization of MQDs. **a** Schematic diagram of hydrothermally preparing MQDs. **b** TEM image of MQDs (inset, particle size distribution of MQDs). **c** High-resolution TEM images of MQDs. **d** AFM topography image of MQDs. **e** Height profile (top) and height distribution (bottom) of MQDs. **f** Survey XPS spectra of MQDs. **g** Adsorption structure of sodium ascorbate on Ti_2_C MXene.

Ti_2_C MXene was synthesized by selective etching of the Al layers from the Ti_2_AlC precursor, as verified by comprehensive characterization including X-ray diffraction (XRD), scanning electron microscopy (SEM), and energy-dispersive spectroscopy (EDS) (Fig. S1–2). Subsequently, high-quality MQDs were fabricated via a hydrothermal treatment of the as-prepared Ti_2_C MXene precursor, wherein sodium ascorbate was introduced as an essential surface-modifying agent to passivate surface states and maintain the integrity of the crystal lattice (Fig. 1a). The as-synthesized MQDs demonstrate exceptional dispersibility and uniform morphological characteristics, possessing an average lateral diameter of 2.269 ± 0.416 nm (Fig. 1b). In the control synthesis without sodium ascorbate, few formations of MQDs were observed in the absence of sodium ascorbate (Fig. S3). High-resolution transmission electron microscopy (HRTEM) imaging confirmed the crystalline structure of the MQDs. The lattice spacing of ∼0.212 nm can be indexed to the (104) plane of Ti_2_C MQDs, supporting preserved local Ti–C ordering (Fig. 1c and S4) [26,27]. Consistently, a weak feature at ∼43.5° is observed in the XRD pattern of the MQDs, corresponding to a d-spacing of ∼0.208 nm, in agreement with the HRTEM results (Fig. S5). Atomic force microscopy (AFM) topography further corroborated their ultrathin profile, indicating a uniform height of 1.120 ± 0.337 nm, consistent with single-layer MXene (Fig. 1d and e) [28].

Furthermore, X-ray photoelectron spectroscopy (XPS) quantitative analysis determined a titanium (Ti) content of 17.80 wt% in the MQDs synthesized with sodium ascorbate—the highest reported value to date for Ti_2_C-based quantum dots (Fig. 1f and S6) [16,17,25,29,30]. To obtain an accurate Ti content of the colloidal dispersion, inductively coupled plasma optical emission spectroscopy (ICP-OES) was conducted and revealed a Ti content of 23.85 wt% (Table S3). These results stand in marked contrast to the significant Ti loss detected in the control experiment, underscoring the efficacy of the present synthetic strategy in maintaining the structural integrity of the Ti_2_C framework throughout the quantum dot formation process.

The critical function of sodium ascorbate in mediating the formation of atomically preserved MQDs was systematically investigated through complementary analysis of its coordination behavior and redox properties. Density functional theory (DFT) calculations revealed a strong binding affinity (−4.92 eV) of ascorbate anions toward Ti-rich edges and defect sites on Ti_2_C MXene (Fig. 1g), indicating the formation of stable coordination complexes. To experimentally corroborate the coordination effect, ascorbic acid, which exhibits a comparable redox potential but deficient coordination capability, was employed as a control ligand. The MQDs synthesized in the presence of ascorbic acid exhibited a photoluminescence (PL) intensity of only 22.8 % relative to the sodium ascorbate group (Fig. S7), and XPS analysis indicated a markedly reduced Ti content of merely 3.0 wt% (Fig. S8), underscoring the indispensability of effective coordination in inhibiting Ti leaching and subsequent oxidative degradation. Furthermore, the redox contribution was evaluated using trisodium citrate as a comparison agent, which provides strong coordination but exhibits negligible reducing activity. The resulting product displayed negligible PL emission (Fig. S7) and comprehensive oxidation of Ti to Ti^4+^ (Fig. S9), confirming the inability to generate fluorescent MQDs in the absence of redox protection. Collectively, these control experiments definitively establish the dual function of sodium ascorbate. Its coordinative action stabilizes Ti species against dissolution, while its reductive activity prevents oxidative degradation, collectively enabling the synthesis of structurally intact and optically active MQDs.

### 2.2 ROS generation properties of MQDs

**Fig. 2:**
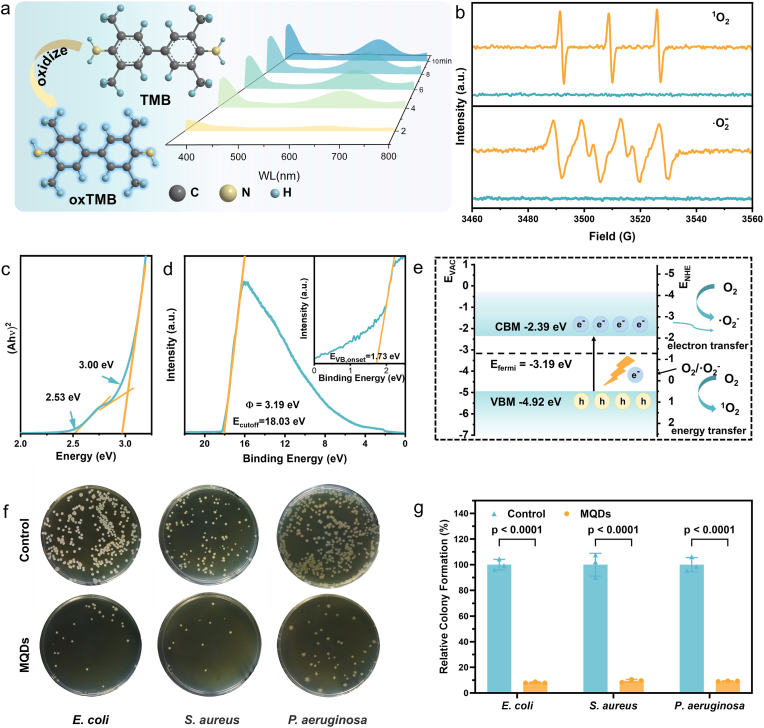
a Schematic illustration and corresponding UV–Vis spectra of MQDs catalyzed oxidation of TMB. **b** ESR spectra of MQDs for the generation of c ^1^O_2_ and d ·O_2_^−^ under 450 nm irradiation (orange) and in the dark (blue). **c** Tauc plot derived from UV–vis absorption spectrum for bandgap estimation. **d** UPS spectra to determine the VBM position of MQDs. **e** Energy band structure of MQDs. **f** Representative optical images of colony-forming units of different viruses after incubating with MQDs and exposing to 450 nm light. Each experiment was repeated independently three times with similar results. **g** Bactericidal efficacy of MQDs characterized by the standard plate counting assay. (n = 3 biologically independent samples; mean ± s.d.).

Benefiting from the well-preserved Ti–C framework, the atomically intact MQDs exhibit distinct optical properties that are directly relevant to their ROS generation performance. UV–vis absorption spectra revealed two characteristic bands at approximately 350 and 450 nm (Fig. S10). Notably, the absorption at 450 nm has not been previously documented in earlier studies on MQDs [16,[22], [23], [24], [25],^29^,^31^,^32^], suggesting a unique electronic structure attributable to the present synthesis strategy. Given the deeper tissue penetration and lower phototoxicity of visible light, 450 nm irradiation was selected to evaluate the ROS generation of MQDs. The overall ROS generation was first verified using 3,3′,5,5′-Tetramethylbenzidine (TMB), where ROS oxidizes TMB to oxTMB, producing a characteristic absorption peak at 652 nm. Under xenon lamp irradiation at 450 nm (0.1 W cm^−2^, 20 min), a strong absorption signal was observed (Fig. 2a), indicating substantial ROS generation by the MQDs. Subsequently, electron spin resonance (ESR) spectroscopy was employed to identify the specific ROS species. With TEMP and BMPO as trapping agents, the characteristic signals of ^1^O_2_ and ·O_2_^−^ appeared under 450 nm irradiation, whereas the dark controls showed negligible responses (Fig. 2b), confirming light-triggered ROS generation.

The above results indicate that MQDs mediate ROS generation through both type I (electron transfer, producing ·O_2_^−^) and type II (energy transfer, generating ^1^O_2_) photodynamic pathways. In contrast to most clinically used organic photosensitizers (e.g., porphyrins, chlorins, phthalocyanines), which predominantly rely on the oxygen-dependent type II mechanism [33], MQDs enable a dual ROS generation mechanism, allowing ROS generation through the less oxygen-dependent type I pathway even under hypoxic conditions. This hypoxia-adaptive property highlights the potential of MQDs for practical biomedical applications, particularly in chronic wound treatment, where oxygen deficiency is prevalent.

To elucidate the ROS generation mechanism, the band structure of the MQDs was analyzed from their absorption spectrum. By transforming the absorption spectra into Tauc plots (Fig. 2c), two optical band gaps of 3.00 eV and 2.53 eV were determined. As the above ROS generation was induced by 450 nm excitation, the bandgap of 2.53 eV, which corresponds closely to the energy of these photons, was selected for subsequent band edge analysis.

The valence band maximum (VBM) was determined using ultraviolet photoelectron spectroscopy (UPS). From the spectra (Fig. 2d), the secondary electron cutoff energy ($E_{\text{cutoff}}=18.03\;\text{eV}$) and the valence band onset energy ($E_{\text{VB},\text{onset}}=1.73\;\text{eV}$) were obtained, enabling the calculation of a VBM value of −4.92 eV according to Equation (1). Based on this value and the bandgap of 2.53 eV, the conduction band minimum (CBM) was then calculated to be −2.39 eV, as illustrated in the constructed energy level diagram (Fig. 2e). Given that the photoexcited electrons of the MQDs possess sufficient reducing power relative to the O_2_/ ·O_2_^−^ reduction potential −0.33 V vs NHE), they have adequate energy to initiate the formation of ·O_2_^−^ via an electron transfer pathway (Type I). Furthermore, the bandgap energy is also sufficiently large relative to the singlet-triplet energy splitting of molecular oxygen (∼0.98 eV) [34], allowing the photoexcited MQDs to generate ^1^O_2_ via an energy transfer pathway (Type II).

Equation 1 $$E_{\text{VBM}}=h\nu ‐\left(E_{\text{cutoff}}‐E_{\text{VB},\text{onset}}\right)$$

Furthermore, the MQDs exhibited PL characteristics that provide additional support for the proposed band structure model (Fig. S11). Under excitation wavelengths ranging from 300 to 390 nm, a blue emission was observed, which redshifted from 410 to 440 nm. This behavior is consistent with a radiative transition associated with the 3.00 eV band gap, attributed predominantly to surface-state-mediated recombination. Conversely, when excited within the 440–490 nm range, a green emission band centered at approximately 535 nm was detected, aligning with the intrinsic band-edge recombination corresponding to the 2.53 eV optical transition.

Consistently, the wavelength-dependent ROS generation of MQDs further substantiates the distinction between intrinsic band-edge excitation and surface-state excitation. As shown in Fig. S12, ^1^O_2_ was detected under 365–520 nm irradiation using singlet oxygen sensor green (SOSG) as the fluorescent probe. The weaker ^1^O_2_ signals were detected under 365–395 nm irradiation, although the MQDs exhibit higher absorbance in the UV region. This mismatch arises because UV excitation primarily addresses surface/defect states that dissipate energy nonproductively, whereas visible excitation accesses intrinsic band-edge states that more effectively channel energy into ^1^O_2_ formation. These results indicate that the photophysics of the MQDs is dominated by intrinsic band edge processes rather than defect-mediated emissive pathways, which underlie their efficient ROS generation under visible light. To define the practical onset of ROS generation, an irradiance-dependent experiment was conducted. As the irradiance increased up to 1 mW cm^−2^, the ^1^O_2_ signal rose continuously and exceeded the 3σ noise threshold (Fig. S13). This criterion identifies 1 mW cm^−2^ as the onset irradiance for detectable ROS generation.

This dual-emission signature was also identified in endothelial cells (ECs) (Fig. S14), confirming the successful cellular internalization of the MQDs and the preservation of their optical functionality within a biological context. The combination of this demonstrated bio-compatibility and efficient ROS generation under visible light irradiation motivated further investigation into the application of MQDs as a novel photodynamic biomedical platform. Wound microenvironments contain oxidants such as H_2_O_2_ and MPO-derived HOCl, and often exhibit mildly acidic conditions. To ensure the chemical and photophysical stability of the MQDs under these perturbations, the absorption and emission behaviors were examined. Although the ligand-related 250–300 nm absorption band displays the expected protonation-induced variation, the intrinsic 300–500 nm visible-light absorption and PL features of the MQDs remain stable under all tested conditions (Fig. S15–S16). In addition, the characteristic absorption band of the MQDs remained unchanged over a 7-day monitoring period (Fig. S17), further confirming their robust optical stability under standard storage and handling conditions.

These findings indicate that MQDs are well preserved in biologically relevant oxidative and mildly acidic environments, supporting sustained visible-light-driven ROS generation. Therefore, their ROS-mediated antibacterial activity was evaluated *in vitro* against common pathogenic bacteria, including *Escherichia coli* (*E. coli*), *Staphylococcus aureus* (*S. aureus*), and *Pseudomonas aeruginosa* (*P. aeruginosa*), which are frequently isolated from diabetic wound infections [35]. As shown in Fig. 2f, light irradiation in the presence of MQDs markedly reduced bacterial viability, as quantified by colony-forming units (CFUs). At a stringent high inoculum, 80 μg mL^−1^ MQDs achieved >90 % killing against *E. coli*, *S. aureus*, and *P. aeruginosa* (Fig. 2g). To provide standardized antibacterial metrics, the minimum inhibitory concentration (MIC) was further determined by broth microdilution using a standard inoculum. The MIC values were 80 μg mL^−1^ for *E. coli* and *P. aeruginosa*, and 160 μg mL^−1^ for *S. aureus* (Table 1, Fig. S18–S20). The minimum bactericidal concentration (MBC), defined as the lowest concentration yielding ≥99.9 % killing upon agar plating from non-turbid wells, was 160 μg mL^−1^ for *E. coli* and *P. aeruginosa*, and 320 μg mL^−1^ for *S. aureus* (Table 1, Fig. S21–S23). Collectively, these results demonstrate that MQDs enable robust light-activated antibacterial activity and achieve bactericidal outcomes at higher doses across all three representative pathogens.

**Table 1:** MIC and MBC values of MQDs against different bacterial strains.

Bacteria	MIC (μg mL^−1^)	MBC (μg mL^−1^)
*E. coli*	80	160
*P. aeruginosa*	80	160
*S. aureus*	160	320

### 2.3 ROS scavenging properties of MQDs

**Fig. 3:**
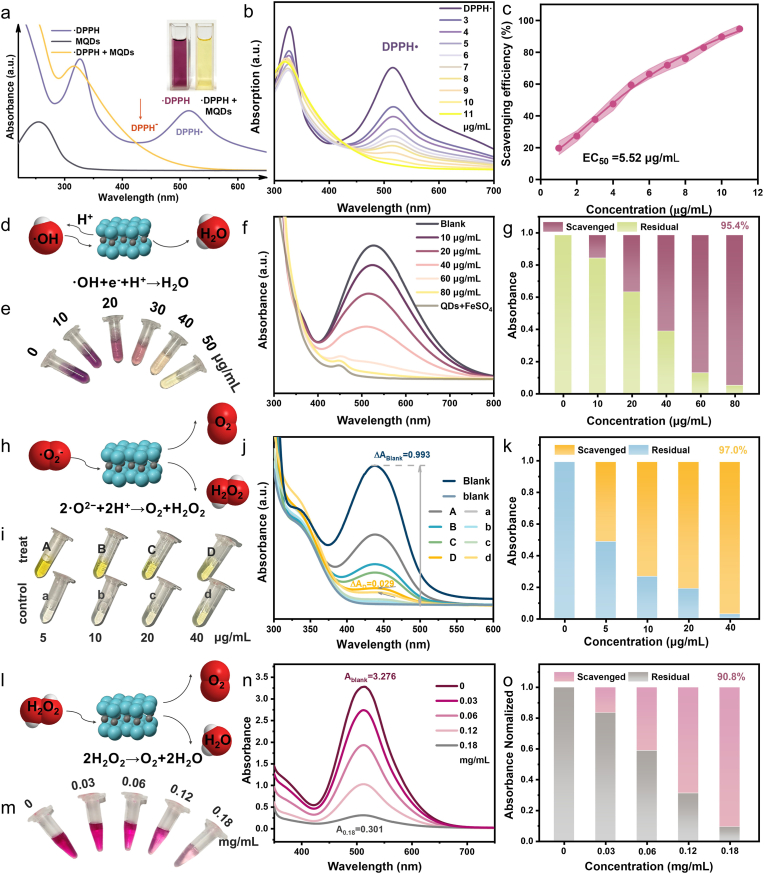
a UV–Vis spectra of ·DPPH and MQDs. **b** UV–Vis spectra of ·DPPH after the addition of different concentrations of MQDs. **c** Dose–response curve for ·DPPH scavenging by MQDs (n = 3, mean ± s.d.) **d**. Schematic diagram of ·OH scavenging. Variation of **e** color, **f** UV–Vis spectra and **g** Scavenging efficiency of ·OH at different concentrations of MQDs. **h** Schematic diagram of ·O_2_^−^ scavenging. Variation of **i** color, **j** UV–Vis spectra and **k** Scavenging efficiency of ·O_2_^−^ at different concentrations of MQDs. **l** Schematic diagram of H_2_O_2_ scavenging. Variation of **m** color, **n** UV–Vis spectra and **o** Scavenging efficiency of H_2_O_2_ at different concentrations of MQDs.

Besides light-activated ROS generation, MQDs also exhibit a pronounced intrinsic reductive capability under dark conditions. This light-controlled bidirectional ROS behavior is further corroborated by reversible light on–off oxidation–reduction cycling experiments (Fig. S24). Then, the antioxidant capacity of MQDs was assessed by the ·DPPH assay, in which ·DPPH reduction is indicated by a decrease in the characteristic absorbance at 517 nm. The addition of MQDs (10 μg mL^−1^) resulted in a significant decrease in ·DPPH absorbance within seconds (Fig. 3a). This effect was dose-dependent, as evidenced by the gradual diminishment of the 517 nm peak with increasing MQDs concentration (Fig. 3b). The EC_50_ value [36] (the concentration required to scavenge 50 % ·DPPH) of MQDs was calculated to be 5.52 μg mL^−1^, which was standardized to 43.53 ng (MQDs) nmol^−1^ (·DPPH) (Fig. 3c). This value is comparable to quercetin, one of the most potent natural antioxidants [37], while markedly lower than the values of typical nanomaterials, including Ag NPs, CeO_2_ NPs, and carbon quantum dots, which are usually reported in the tens to hundreds of μg mL^−1^ range [[38], [39], [40]].

Then, the origin of the excellent antioxidant capability of MQDs was investigated. Scavenging of the DPPH radical is known to proceed through both hydrogen atom transfer (HAT) and single electron transfer (SET) mechanisms, with the predominant pathway being determined by the specific antioxidant. The rapid reaction kinetics occurring on a second timescale suggest that the HAT mechanism is dominant, as direct hydrogen abstraction typically occurs more rapidly than electron transfer processes, which involve the formation of intermediate species [41] (Fig. S25). Concurrently, the appearance of new absorption peaks at 380 nm and 438 nm in the UV–vis spectrum, corresponding to the formation of DPPH^+^ and DPPH^−^ species respectively [42], provides clear evidence for the concurrent operation of the SET pathway. Therefore, the superior antioxidant performance of the MQDs can be attributed to their abundant surface functional groups, which act as effective hydrogen donors (Fig. S26), in combination with unsaturated Ti atoms at the edges that facilitate efficient electron donation [43,44].

Given the dual capacity for hydrogen donation and electron transfer, the antioxidant performance of MQDs was further evaluated under physiologically relevant conditions against three representative endogenous ROS, including ·OH, ·O_2_^−^, and H_2_O_2_. The detection of •OH was conducted using salicylic acid as a molecular probe, which forms the chromogenic product 2,3-dihydroxybenzoic acid exhibiting a characteristic absorption peak at 520 nm. Owing to their synergistic capacity to donate both electrons and hydrogen atoms, the MQDs effectively neutralized •OH, converting it into water (Fig. 3d). This scavenging effect was corroborated by a visible fading of the solution coloration and a corresponding decrease in absorbance at 520 nm (Fig. 3e and f). At a concentration of 80 μg mL^−1^, the MQDs demonstrated a •OH scavenging efficiency exceeding 95 % (Fig. 3g).

The O_2_^−^ scavenging activity was assessed using the water-soluble tetrazolium salt WST-1 (2-(4-iodophenyl)-3-(4-nitrophenyl)-5-(2,4-disulfophenyl)-2H-tetrazolium), which undergoes reduction by O_2_^−^ to generate a formazan product exhibiting a characteristic absorption peak at 450 nm. The MQDs facilitate the dismutation of O_2_^−^ into oxygen and hydrogen peroxide via hydrogen atom donation (Fig. 3h). This reaction was evidenced by a pronounced fading of the formazan coloration and a corresponding reduction in absorbance at 450 nm (Fig. 3i and j). At a concentration of 40 μg mL^−1^, the MQDs exhibited a high O_2_^−^ scavenging efficiency of 97.0 % (Fig. 3k).

The quantification of H_2_O_2_ was performed using a peroxidase-coupled chromogenic assay, monitored by its characteristic absorbance at 520 nm. The MQDs could catalyze the decomposition of H_2_O_2_ into O_2_ and H_2_O (Fig. 3l), resulting in the fading of the chromogenic substrate and the decrease in its absorbance at 520 nm (Fig. 3m and n). At a concentration of 0.18 mg mL^−1^, the MQDs demonstrated a H_2_O_2_ scavenging efficiency of 90.80 % against a 1 mM H_2_O_2_ solution (Fig. 3o), far exceeding the H_2_O_2_ levels typically observed in wound environments (100–250 μM) [45]. These findings highlight the superior antioxidant capacity of MQDs, combining broad-spectrum ROS scavenging, mechanistic flexibility, and high scavenging efficiency at low dosages.

### 2.4 Biocompatibility of MQDs

**Fig. 4:**
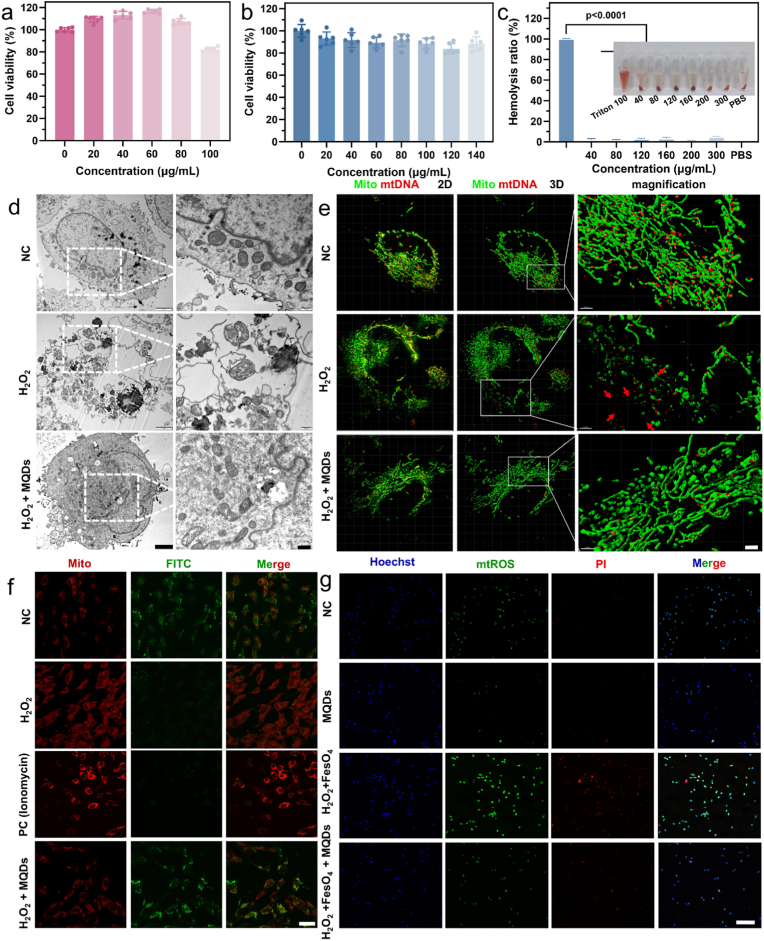
Cell viability of **a** ECs and **b** HaCaT after treatment with MQDs for 24 h (n = 6 biologically independent samples; mean ± s.d.). **c** Hemolytic activity of red blood cells after incubation with MQDs for 4 h (n = 6 biologically independent samples; mean ± s.d.). **d** TEM images of mitochondria after various treatments. Scale bars are 2 μm (left) and 500 nm (right). **e** His-SIM images of ECs after various treatments. Scale bar is 20 μm. **f** CLSM images of ECs after various treatments stained with Mitto/FITC. Scale bar is 40 μm **g** CLSM images of ECs after various treatments stained with Hoechst/mtROS/PI. Scale bar is 250 μm.

Given the potent antioxidant performance of MQDs under physiologically relevant conditions, their biosafety was next systematically evaluated to ensure suitability for biomedical applications. *In vitro* cytotoxicity was assessed using CCK-8 assays, which showed that cell viability remained above 90 % even at 80 μg mL^−1^ MQDs (Fig. 4a and b). The half-maximal inhibitory concentration (IC_50_) for HaCaT cells was determined to be 243.33 μg mL^−1^ (Fig. S27). Hemolysis testing further demonstrated excellent blood compatibility, with hemolysis ratios staying well below the 5 % safety threshold up to 300 μg mL^−1^ (Fig. 4c). Fluorescence microscopy showed only sporadic PI-stained nuclei at 80 μg mL^−1^, and quantitative analysis revealed that apoptosis increased by ∼9.6 % relative to the control (Fig. S28). To profile in vivo exposure, biodistribution was assessed by ICP–MS quantification of Ti in plasma and major organs at 24 h and 7 days post-administration. Organ burdens were expressed as the percentage of injected dose (%ID). The distribution followed a reticuloendothelial system dominated profile, with the liver as the primary deposition site. Notably, the hepatic burden remained a minor fraction of the injected dose, less than 6 %ID, while the spleen and kidney each accounted for less than 1 %ID, and plasma Ti levels remained low (Fig. S29, Table S4). No statistically significant differences were observed between the two time points, suggesting no progressive accumulation over this time window. Collectively, these results support the favorable *in vitro* hemocompatibility and cytocompatibility of MQDs and provide in vivo distribution information relevant to their biomedical use.

### 2.5 MQDs alleviate oxidative stress *in vitro*

To investigate the intracellular antioxidant performance of MQDs, particular attention was given to their ability to preserve mitochondrial integrity under oxidative stress conditions. Mitochondria are central to cellular metabolism and ROS regulation, and are among the primary targets of oxidative damage. Mitochondrial ultrastructure was examined using TEM and high-resolution structured illumination microscopy (His-SIM). In healthy cells, mitochondria maintain an elongated, tubular morphology (Fig. 4d) with densely packed cristae, and mitochondrial nucleoids (red, DNA-protein complexes) are uniformly distributed between cristae folds (green) (Fig. 4e) [46]. Following exposure to H_2_O_2_, mitochondrial networks underwent fragmentation into rounded structures, accompanied by disorganization of cristae and aggregation of mitochondrial DNA (mtDNA), indicative of substantial structural disruption. Notably, co-treatment with MQDs preserved mitochondrial architecture, suppressed mtDNA leakage, and maintained cristae integrity, underscoring the protective effect against oxidative stress.

To further assess the protective effect of MQDs on mitochondrial function, the status of the mitochondrial permeability transition pore (mPTP) was assessed using calcein-AM/CoCl_2_ staining. In this assay, calcein-AM accumulates within mitochondria and produces green fluorescence upon hydrolysis; however, this fluorescence is quenched by Co^2+^ upon mPTP opening. Both ionomycin and H_2_O_2_ were observed to induce mPTP opening, resulting in a pronounced loss of fluorescence. In contrast, cells co-treated with H_2_O_2_ and MQDs exhibited sustained fluorescence intensity, suggesting that MQDs attenuated the oxidative stimuli and preserved mPTP integrity (Fig. 4f).

The capability of MQDs to mitigate cellular oxidative damage was determined in ECs. The Fenton reaction, mediated by Fe^2+^ and H_2_O_2_, generates extremely toxic ·OH, resulting in massive cell death. Compared with the NC/MQDs group, treatment with Fenton's reagent induced a marked upregulation of mitochondrial ROS (mtROS), subsequently triggering significant apoptosis(Fig. 4g). In particular, MQDs effectively alleviate oxidative stress and notably attenuate H_2_O_2_-induced oxidative damage, thus conferring protection against cell death. Consistent results were observed in HaCaT cells, wherein H_2_O_2_ stimulation elicited pronounced intracellular oxidative stress. By contrast, MQDs treatment resulted in a dose-dependent reduction in oxidative stress levels, with higher concentrations yielding more substantial protective effects (Fig. S30).

### 2.6 MQDs modulate the immune response

**Fig. 5:**
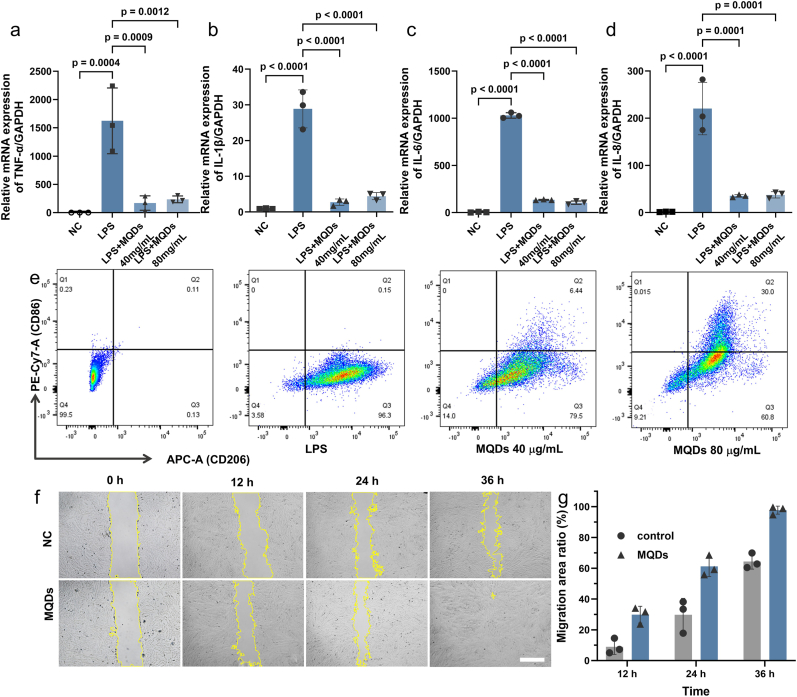
a TNF-*α*, **b** IL-1*β*, **c** IL-6, and **d** IL-8 contents in J774a.1 cells treated with LPS, LPS + MQDs (40/80 μg mL^−1^) for 24 h (n = 3 biologically independent experiments; mean ± s.d.). **e** CD86/CD206 flow cytometry of J774a.1 cells treated with LPS, LPS + MQDs (40/80 μg mL^−1^) for 24 h **f** Representative images of ECs migration. Scale bar is 100 μm **g** Quantitative migration area ratio during the observation time. (n = 3 independent samples; mean ± s.d).

Given the tight interplay between oxidative stress and inflammation, the anti-inflammatory properties of MQDs were further investigated to evaluate their broader therapeutic potential. This assessment was conducted using a lipopolysaccharide (LPS)-induced inflammation model in J774A.1 macrophage cells. As illustrated in Fig. 5a–d, LPS stimulation resulted in a marked upregulation of pro-inflammatory cytokines, including tumor necrosis factor-α (TNF-*α*), interleukin-1β (IL-1*β*), interleukin-6 (IL-6), and interleukin-8 (IL-8). Treatment with MQDs significantly suppressed the secretion of these cytokines, indicating their capability to attenuate cellular inflammatory responses.

Since macrophage polarization is highly important in regulating inflammation and tissue repair [47,48], the potential of MQDs to influence macrophage phenotypic switching was further evaluated. Flow cytometry revealed that MQDs markedly reduced CD86 expression (M1 phenotype marker) while enhancing CD206 expression (M2 phenotype marker) (Fig. 5e), suggesting a phenotypic shift from pro-inflammatory M1 to pro-reparative M2 macrophages [49,50]. Collectively, these results suggest that MQDs not only suppress cytokine secretion but also actively direct macrophage polarization, thereby facilitating the resolution of inflammation.

The process of wound healing involves cell activation and recruitment, in which cell migration plays an important role. The promotion ability for cell migration of MQDs was evaluated by the cell scratch assay (Fig. 5f and g). At 12 h post-scratch, the cell migration area ratio in the MQDs group reached 62 %, significantly higher than the 30 % observed in the control group. By 36 h, the cell migration area ratio in the MQDs group approached nearly 100 %, markedly exceeding that of the control. These results indicate that MQDs not only alleviate the inflammatory burden but also actively enhance cell migration, thereby contributing to the re-epithelialization and tissue repair process during wound healing.

### 2.7 Preparation and characterization of MQDs@Col-SA MN

**Fig. 6:**
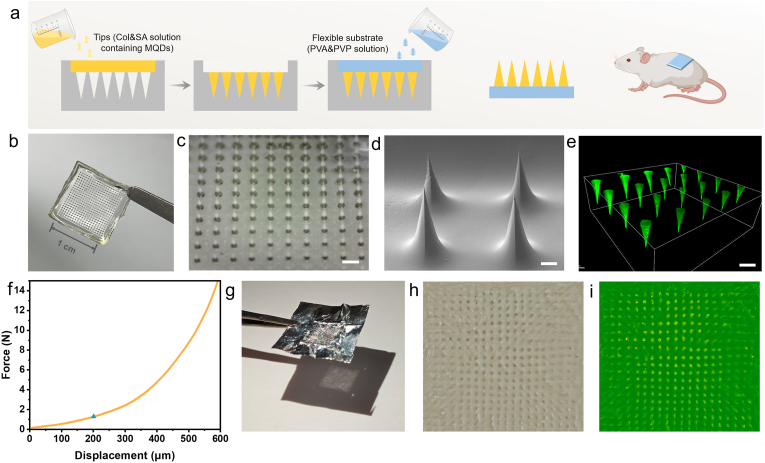
Fabrication of MQDs@Col-SA MN. **a** The fabrication process of MQDs@Col-SA MN. **b** Digital camera image of MQDs@Col-SA MN. **c** Stereo-microscopic image of MQDs@Col-SA MN. Scale bar is 500 μm **d** SEM image of MQDs@Col-SA MN. Scale bar is 100 μm. **e** Fluorescence microscopy image of MQDs@Col-SA MN. Scale bar is 300 μm. **f** Force-displacement curves of MQDs@Col-SA MN. **g** The insertion ability of MQDs@Col-SA MN. **h** and **i** Micropore array formed by dyed needle tips.

The capabilities of MQDs to modulate ROS levels, alleviate inflammation, and promote cell migration endow them with significant potential for biomedical applications, particularly in wound therapy. To enable targeted delivery of MQDs to wound sites, they were encapsulated within a polymer network to form needle tips capable of direct tissue penetration (designated as MQDs@Col-SA MN). A two-step casting methodology was employed (Fig. 6a), wherein collagen (Col) and sodium alginate (SA) were utilized as the matrix materials for MQDs loading in the tip compartment. The pedestal of microneedle was fabricated from polyvinyl alcohol(PVA) and polyvinyl pyrrolidone(PVP), conferring mechanical flexibility and cutaneous adhesiveness. Macroscopic evaluation of MQDs@Col-SA MN displayed a regularly arranged array with sharply tapered needles (Fig. 6b and c). SEM further illustrated the microneedle morphology, confirming a quadrangular pyramid structure with intact tip geometry(Fig. 6d). Additionally, confocal microscopy confirmed the homogeneous distribution of MQDs within the needle tips, as evidenced by uniform fluorescence emission (Fig. 6e).

To ensure accurate delivery of MQDs into wound tissue, the microneedle tips of MQDs@Col-SA MN need to be implanted into the skin without bending or breaking. Therefore, the mechanical properties of MQDs@Col-SA MN system were first evaluated. As depicted in Fig. 6f, with a 200 μm displacement, the needle strength of MQDs@Col-SA MN has an approximate force of 1.26 N, substantially exceeding the 0.4 N of force required to puncture the skin [51]. Penetration capability was further assessed using a metal foil substrate. Inserting the MQDs@Col-SA MN into the foil, it can be seen that the foil was left with a complete array of micropores (Fig. 6g). Furthermore, the skin insertion performance was validated *ex vivo* on porcine skin. Following application of a methylene blue-loaded microneedle patch, a complete pattern of blue micropores was observed on the skin surface (Fig. 6h and i), demonstrating successful penetration of the stratum corneum. These results collectively demonstrate that MQDs@Col-SA MN possesses suitable mechanical strength and structural robustness for effective transdermal delivery.

### 2.8 MQDs@Col-SA MN promotes diabetic wound healing

**Fig. 7:**
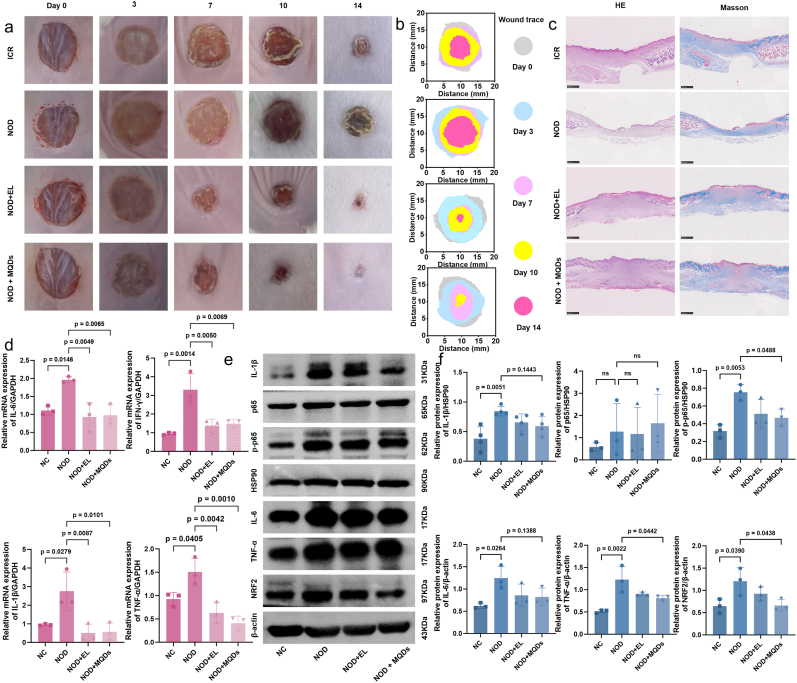
Promoting diabetic wound healing by MQDs@Col-SA MN. **a** Photographs of representative wounds from different groups at different time points. **b** Schematic diagram of the wound-healing process in different groups. **c** H&E and Masson's trichrome staining images of wound samples treated with different groups on the 14th day. Each experiment was repeated five times independently with similar results. **d** The mRNA levels of inflammatory factors (IL-6, IFN-*γ*, IL-1*β*, and TNF-*α*) of the different groups (n = 3 biologically independent experiments; mean ± s.d.). **e** The protein expressions and **f** relative protein expression of inflammatory factor (IL-1*β*, p65, p-p65, IL-6, and TNF-*α*) and oxidative stress factor (NRF2) by WB (n = 3 biologically independent experiments; mean ± s.d.).

To evaluate the in vivo therapeutic efficacy of MQDs@Col-SA MN, both nonobese diabetic (NOD) and Institute of Cancer Research (ICR) mice were employed as wound models. Three groups were established for comparison, healthy ICR mice, untreated diabetic NOD mice, and NOD mice treated with ethacridine lactate (EL), a known antimicrobial and anti-inflammatory agent with associated biotoxicity (Fig. S31). As expected, wound healing was significantly impaired in NOD mice compared to ICR controls. While EL treatment partially improved healing in the diabetic group, MQDs@Col-SA MN group exhibited superior wound closure efficiency. Prominent wound contraction and scab formation were visible as early as day 7 in the MQDs-treated mice, whereas the NOD group exhibited a tendency toward further wound expansion (Fig. 7a and b). By Day 10, wounds in the MQDs@Col-SA MN group were nearly closed, while those in the ICR and NOD groups showed minimal reduction in wound area. Quantitative analysis confirmed residual wound areas of 70.6 % in the ICR group, 90.6 % in the NOD group, 23.3 % in the NOD + EL group, and only 9.8 % in the MQDs@Col-SA MN group (Fig. S32). At the end of the observation period (Day 14), wounds in the MQDs@Col-SA MN group were almost completely healed, whereas those in the NOD group still retained more than 50 % of the original wound area.

Histological analysis via H&E and Masson's trichrome staining (Fig. 7c) revealed marked disparities in tissue regeneration across the experimental groups. By day 14, skin sections from the ICR group exhibited an intact epidermis, dense and well-organized collagen fibers, and minimal inflammatory cell infiltration, indicative of normal dermal architecture and a homeostatic microenvironment (Fig. S33). In contrast, the NOD group showed severe tissue disorganization, with a thinned epidermis, fragmented and sparse collagen fibers, and extensive infiltration of inflammatory cells in the dermis, indicating impaired matrix remodeling and persistent inflammation. The NOD + EL group showed moderate restorative effects, including enhanced collagen deposition and moderately restored dermal structure; yet notable inflammatory infiltration persisted. Remarkably, the NOD + MQDs group displayed the most pronounced recovery, characterized by a continuous epidermis, densely aligned collagen bundles, compact dermal tissue, and significantly reduced inflammatory cell infiltration. These results suggest that MQDs@Col-SA MN not only enhance extracellular matrix remodeling through promoted collagen regeneration and dermal maturation, but also effectively suppress local inflammation, addressing both the structural damage and chronic inflammatory milieu associated with diabetic wound pathology.

To further assess the inflammatory microenvironment and oxidative stress at the wound site, quantitative real-time PCR (qRT-PCR) was performed to measure the expression levels of key pro-inflammatory cytokines, including IL-6, IL-1*β*, TNF-*α*, and interferon-*γ* (IFN-*γ*) (Fig. 7d). As expected, wound tissues from NOD mice exhibited elevated expression of all evaluated cytokines relative to healthy controls. Both EL and MQDs@Col-SA MN treatments markedly downregulated the transcription of these inflammatory mediators. Notably, MQDs@Col-SA MN reduced IL-6, IL-1*β,* and TNF-*α* expression to levels below those observed in the ICR group, indicating a potent modulatory effect on both inflammation and oxidative stress under diabetic wound conditions.

To substantiate the regulatory role of MQDs in modulating the wound microenvironment, Western blot analysis was employed to examine protein expression profiles related to inflammatory and oxidative stress pathways (Fig. 7e and f). In the diabetic NOD group, a marked upregulation of pro-inflammatory cytokines was observed, including IL-1*β*, IL-6, and TNF-*α*, accompanied by enhanced activation of the NF-*κ*B signaling pathway, as indicated by elevated levels of both total p65 and phosphorylated p65 (p-p65). Simultaneously, increased expression of NRF2 suggested a compensatory response to excessive oxidative stress. Although EL treatment partially reduced the expression of these pathological markers, administration of MQDs@Col-SA MN resulted in a more pronounced suppression across all indicators. These molecular-level observations corroborate the histological results and demonstrate the capacity of rationally engineered nanomaterials to reprogram the hostile wound microenvironment toward a regenerative state. Together, these findings confirm that MQDs@Col-SA MN exceeds the efficacy of conventional therapeutic agents by simultaneously targeting both upstream initiators and downstream effectors of diabetic wound pathology.

In addition to the therapeutic benefits, systemic safety was evaluated after the wound-healing treatment. Serum alanine aminotransferase (ALT), aspartate aminotransferase (AST), blood urea nitrogen (BUN), and creatinine (CREA) levels remained within normal physiological ranges, and H&E staining of the major organs showed no detectable inflammation or tissue damage (Fig. S34–35). These results indicate that MQDs@Col-SA MN does not induce off-target toxicity during the therapeutic period, highlighting its suitability for further translational development.

## 3 Conclusions

This study developed a coordination-reduction strategy to synthesize atomically preserved Ti_2_C MQDs, overcoming the long-standing challenges of Ti leaching and oxidative degradation. The MQDs exhibited enhanced crystallinity and preserved Ti sites, minimizing defects while preserving redox-active centers, thereby endowing them with dual ROS regulation capability. By preserving intrinsic band-structure features, MQDs extended photodynamic ROS generation into the visible region, enabling efficient antibacterial activity against pathogenic bacteria under visible-light excitation. Meanwhile, abundant unsaturated Ti sites and surface functional groups endowed the MQDs with hydrogen-donating and electron-transfer capacities, conferring exceptional ROS scavenging performance that mitigated oxidative stress, suppressed inflammation, and promoted M2 macrophage polarization. Incorporated into a collagen–alginate microneedle system, MQDs@Col-SA MN further validated the therapeutic efficacy of MQDs in diabetic wound model, achieving ∼80 % smaller wound area than untreated controls at Day 10. This work presents the first demonstration of structurally intact MQDs with adaptive redox regulation, defining a paradigm for light-responsive biomaterials in regenerative medicine.

## 4 Experimental section

### 4.1 Preparation of MQDs@Col-SA MN

Ti_2_C MXene was synthesized via classical acid etching of Ti_2_AlC, followed by repeated washing until neutral. Sodium ascorbate was then introduced as a protective agent to minimize oxidation and decomposition. The stabilized MXene was further processed through hydrothermal treatment and dialysis to obtain MQDs. MQDs were incorporated into a collagen/sodium alginate (Col/SA) network, and microneedles were fabricated using a two-step casting process with PDMS molds, forming 600 μm rectangular pyramid tips on the pedestal of PVA and PVP pedestals. Detailed experimental procedures are provided in the Supplementary Information.

### 4.2 Materials characterizations

XRD patterns were measured by Rigaku Ultima IV X-ray diffractometer. SEM images and EDS images were obtained by Germany ZEISS Gemini SEM 300. TEM images were obtained by JEOL JEM-F200 transmission electron microscope. Fluorescence microscopy images were obtained by Oxford Andor BC43 spinning disk confocal microscope. FT-IR was obtained by Thermo Scientific Nicolet iS5. XPS was obtained by Thermo Scientific K-Alpha photoelectron spectroscopy. Ti content was quantified using ICP–OES by Agilent 720 ES or ICP–MS by Agilent 7200. UV–Vis absorbance spectra were obtained by MAPADA UV6100 spectrophotometer. PL spectrum was obtained by the F-7000FL spectrum analysis instrument. UPS was obtained by Thermo Fisher Scientific ESCALAB XI + ultraviolet photoelectron spectroscopy.

### 4.3 Evaluation of material properties

#### 4.3.1 ROS generation of MQDs

MQDs were evaluated using a TMB chromogenic assay under 450 nm light (0.1 W cm^−2^), with absorbance measured at different time points. ROS species (·OH, ·O_2_^−^, and ^1^O_2_) were detected by ESR (Bruker A300) spectroscopy, using BMPO and TEMP as trapping agents, under the same light irradiation conditions. Detailed experimental procedures are provided in the Supplementary Information.

#### 4.3.2 ROS scavenging of MQDs

The antioxidant properties of MQDs were evaluated by ·DPPH scavenging, ·OH scavenging, ·O_2_^−^ scavenging, and H_2_O_2_ scavenging. Detailed experimental procedures are provided in the Supplementary Information.

#### 4.3.3 Biocompatibility of MQDs

The cytocompatibility was assessed using the CCK-8 assay with ECs and HaCaT cells exposed to various concentrations of MQDs@Col-SA MN for 24 h. Cell viability was determined by absorbance at 450 nm. Hemolysis was evaluated by incubating erythrocytes with MQDs, followed by measuring absorbance at 545 nm to calculate the hemolysis ratio. Detailed experimental procedures are provided in the Supplementary Information.

#### 4.3.4 Antimicrobial properties of MQDs

*E. coli*, *S. aureus*, and *P. aeruginosa,* as model microbes, were inoculated in NB medium and cultured overnight at 37 °C until reaching an exponential growth stage. MQDs was mixed with a diluted bacterial suspension (10^8^ CFU mL^−1^) and then inoculated into NB medium (Hopebio, China). After irradiation under 450 nm blue light, the appropriate amount of bacterial solution was coated with NA medium (Hopebio, China) by gradient dilution, and incubated at 37 °C overnight. Bacteria that underwent irradiation in the absence of MN served as negative controls. Detailed experimental procedures are provided in the Supplementary Information.

#### 4.3.5 Intracellular oxidative stress Alleviation of MQDs

The protective effect of MQDs on oxidative stress was evaluated by assessing mPTP opening and scavenging of H_2_O_2_ and ·OH in ECs. mPTP opening, indicative of mitochondrial damage, was monitored using mitochondrial probes. Additionally, the material's ability to scavenge H_2_O_2_ and ·OH was evaluated by measuring ROS levels using fluorescence-based assays. The fluorescent images were captured by using a Leica MICA confocal microscope. Micrographs of mitochondria were obtained by Hitachi Field emission scanning electron microscope. High-resolution micrographs of mitochondria were conducted on CSR Biotech High Intelligent and Sensitive Structured Illumination Microscope.

#### 4.3.6 Anti-inflammatory properties of MQDs

qRT-PCR (Roche Lightcyler 96) was used to measure the expression of pro-inflammatory cytokines (IL-8, IL-1β, IL-6, TNF-α). Flow cytometry (BD-FACSCanto-plus) was employed to analyze immune cell populations and their activation status. Detailed experimental procedures are provided in the Supplementary Information.

#### 4.3.7 In vivo assessment of diabetic wound healing

The NOD and ICR mice were purchased from Shanghai Slack Laboratory Animal Co., Ltd. [SCXK (Shanghai) 2022–0004]. The mice were housed and cared for at the Experimental Animal Centre of Zhejiang University of Traditional Chinese Medicine [license number: SYXK (Zhejiang) 2021-0012]. All animal procedures were administered and discarded according to the guidelines of the national animal research code and approved by Zhejiang Chinese Medical University Laboratory Animal Research Center (approval number: IACUC-20241118-28).

The ability of the microneedles to accelerate chronic wound healing was assessed by establishing a full-thickness skin wound model in spontaneously diabetic mice. Wound area changes were recorded and analyzed over 14 days. Histological assessments, including H&E and Masson staining, were performed to evaluate tissue regeneration. In addition, qRT-PCR and Western blot analyses were used to characterize the expression of key genes and proteins related to inflammation and tissue repair. Detailed experimental procedures are provided in the Supplementary Information.

## CRediT authorship contribution statement

**Dejia Hu:** Conceptualization, Data curation, Formal analysis, Investigation, Methodology, Writing – original draft. **Tianhao Xia:** Conceptualization, Data curation, Formal analysis, Investigation, Methodology, Writing – review & editing. **Danyang Xiao:** Conceptualization, Data curation, Formal analysis, Investigation, Methodology, Writing – review & editing. **Bufeng Liang:** Conceptualization, Data curation, Formal analysis, Investigation, Methodology, Writing – review & editing. **Yuyi Li:** Conceptualization, Data curation, Formal analysis, Investigation, Methodology, Writing – review & editing. **Jinkun Li:** Conceptualization, Data curation, Formal analysis, Investigation, Methodology, Writing – review & editing. **Zhongliao Zeng:** Conceptualization, Data curation, Formal analysis, Investigation, Methodology, Writing – review & editing. **Jianxiong Ma:** Funding acquisition, Supervision, Writing – review & editing. **Yan Li:** Funding acquisition, Supervision, Writing – review & editing.

## Declaration of competing interest

The authors declare that they have no known competing financial interests or personal relationships that could have appeared to influence the work reported in this paper.

## Data Availability

Data will be made available on request.
