# Supplementary material for: Atomically preserved MXene quantum dots as a redox-responsive nanoplatform for light-controlled bidirectional ROS engineering
Source: Mater Today Bio. 2026 Jan 28;37:102864. doi: 10.1016/j.mtbio.2026.102864 (PMC12887099; doi:10.1016/j.mtbio.2026.102864)
Supplement: Multimedia component 1 [file mmc1.pdf]

Supplementary Material

Atomically Preserved MXene Quantum Dots as a Redox-Responsive Nanoplatform for Light-Controlled Bidirectional ROS Engineering

Dejia Hu[a], Tianhao Xia[a], Danyang Xiao[a], Bufeng Liang[a], Yuyi Li[b], Jinkun Li[b], Zhongliao Zeng[b], Jianxiong Ma[c, d, *] and Yan Li[a, *]

[a] School of Material Science and Engineering, University of Science and Technology Beijing, Beijing 100083, P.R. China

[b] The Second School of Clinical Medicine, Zhejiang Chinese Medical University, Hangzhou 310053, P.R. China

[c] Department of Nephrology, the First Affiliated Hospital of Zhejiang Chinese Medical University (Zhejiang Provincial Hospital of Traditional Chinese Medicine), Hangzhou 310006, P.R. China

[d] Zhejiang Key Laboratory of Research and Translation for Kidney Deficiency-Stasis-Turbidity Disease, Hangzhou 310000, P.R. China

*Corresponding author. liyan2011@ustb.edu.cn and daxiong1990@zcmu.edu.cn

This supplemental file includes:

Experimental Section

Table 1-4

Fig. S1-S35

Experimental Section

Materials

Layered ternary titanium aluminum carbide (Ti_2AlC, 200 mesh, >98% phase purity) was purchased from Laizhou Kai Kai Ceramic Materials Co., Ltd. (Laizhou, China). HCl, salicylate, and H_2O_2 (30%) were purchased from China National Medicines Corporation Ltd. (Beijing, China). $FeSO_4 \cdot 7H_2O$ was purchased from Beijing Chemical Works (Beijing, China). ·DPPH and Superoxide Dismutase (SOD) activity assay kit (WST-1) were purchased from Beijing Solarbio Science & Technology Co., Ltd. (Beijing, China). LiF and Sodium alginate (SA, 90%) were purchased from Shanghai Macklin Biochemical Co., Ltd. (Shanghai, China). Sodium ascorbate and Collagen (Col) were purchased from Shanghai Boer Chemical Reagent Co., Ltd. (Shanghai, China). Polyvinyl alcohol (PVA) and polyvinyl pyrrolidone (PVP) were purchased from Shanghai Aladdin Biochemical Technology Co., Ltd. (Shanghai, China). Catalase Assay Kit S0051, Mitochondrial Permeability Transition Pore (MPTP) Assay Kit C2009S were purchased from Beyotime Biotech lnc. (Shanghai, China). PK Mito Red was purchased from Guangzhou Computational Super-Resolution Biotech Co., Ltd. (Guangdong, China). Chemicals in this study were analytical reagent grade and used as received. Ultrapure water was used in all experiments.

Preparation of MQDs

Ti_2C MXene was prepared using the classical acid etching method. 20 mL mixed solution of HCl and LiF was configured, where the F- concentration was 1.20 M and the H+ concentration was 6.00 M. Then 1 g MAX phase precursor Ti_2AlC powder was slowly immersed at room temperature and magnetically stirred for 36h. After acid etching by removing the Al layers, the suspension was centrifuged at 5000 rpm and washed and several cycles using deionized water until the pH of the supernatant reached higher than 5.0.

The formation energies for bare MXene are mostly negative in the water stability region, suggesting a spontaneous formation of vacancies at large quantities, and possible material decomposition, under hydrothermal conditions [1]. Therefore, sodium ascorbate was added as a protection agent to minimize the oxidation and decomposition of Ti_2C MXene. 0.30 g of Ti_2C MXene powder was mixed with 20 mL of sodium ascorbate solution (3.6 mg mL^{-1}) and sonicated for 5 min to make it well dispersed. The suspension was stirred at room temperature for 10 h to allow sodium ascorbate to coordinate with the surface of Ti_2C MXene nanosheets. The suspension was then hydrothermally

treated at 120 °C for 6 h. After cooling to room temperature, the supernatant was collected. Then, the collected supernatant was dialyzed against deionized water (MWCO 500 Da) for 24 h, with the dialysate refreshed every 3 h.

Inductively coupled plasma optical emission spectrometry (ICP–OES) measurement of Ti content

For analysis, 1.0 mL of the MQDs dispersion was digested in freshly prepared aqua regia until complete dissolution, followed by dilution to 10.0 mL with ultrapure water. The Ti concentration was obtained from the calibration curve using Ti standards, and the final Ti mass fraction of the MQDs was calculated based on the dried solid content (80 μg mL^{-1}).

DFT calculations

First-principles calculations were carried out using the Vienna Ab initio Simulation Package (VASP). The exchange–correlation interactions were treated using the Perdew–Burke–Ernzerhof (PBE) functional within the framework of generalized gradient approximation (GGA). The projector augmented wave (PAW) method was applied to describe core–valence electron interactions. A kinetic energy cutoff of 400 eV was used for the plane-wave basis set. Brillouin zone sampling was performed using a 2 × 2 × 1 Monkhorst–Pack k-point mesh. Structural relaxations were conducted until the total energy and atomic forces converged to less than 1.0×10^{-4} eV and 0.05 eV $Å^{-1}$, respectively. Dispersion corrections were included via the DFT-D3 scheme to account for van der Waals interactions.

The adsorption energy (E_{ads}) is defined as:

$$E_{ads}=E_{complex}-E_{substrate}-E_{adsorbate}$$

where substrate and adsorbate refer to the substrate surface and adsorbate molecule, respectively.

ROS generation of MQDs.

3,3',5,5'-Tetramethylbenzidine (TMB) chromogenic assay. The ability of MQDs to generate ROS to oxidize substrates was assessed by TMB under 450 nm light (0.1 W cm^{-2}, 20 min). The TMB solution (2 mM) was added into all samples and incubated for different time intervals (0, 2, 4, 6, 8, and 10 min), and the corresponding absorbance was recorded using by UV–vis spectrophotometer.

ROS detection by Electron spin resonance. BMPO was used to trap ·OH and $·O_2^-$, and TEMP was used to trap $^1O_2$. Specifically, MQDs (80 μg mL^{-1}) were mixed with BMPO (20 mM) or TEMP (50

mM). The mixture was irradiated at 450 nm (0.1 W cm^{-2}) for 20 min. EPR spectra were acquired immediately after irradiation.

Wavelength- and intensity-dependent ROS generation

ROS generation was evaluated using singlet oxygen sensor green (SOSG) as the probe. MQDs were mixed with SOSG at a final concentration of 2.5 μM. For wavelength-dependent measurements, the MQDs/SOSG solutions were irradiated with light of different wavelengths at a fixed power density of 20 mW cm^{-2} for 20 min, followed by fluorescence recording of SOSG. For light-intensity-dependent measurements, irradiation was performed at a constant wavelength under different light intensities for 20 min, while all other conditions were kept unchanged.

Antimicrobial properties of MQDs.

To evaluate the antibacterial ability of MQDs, *Escherichia coli (E. coli), Staphylococcus aureus (S. aureus),* and *Pseudomonas aeruginosa (P. aeruginosa)*, as model microbes, were inoculated in NB medium and cultured overnight at 37 °C until reaching an exponential growth stage. MQDs was mixed with a diluted bacterial suspension (10^8 CFU mL^{-1}) and then inoculated into NB medium (Hopebio, China). After irradiation under 450 nm blue light (50 mW cm^{-2}, 40 min), the appropriate amount of bacterial solution was coated with NA medium (Hopebio, China) by gradient dilution, and incubated at 37 °C overnight. Bacteria that underwent irradiation in the absence MQDs served as control. The antibacterial activities were calculated with the following equation:

$$\text{antibacterial activities (\%)}=\frac{N}{N_0}\times 100\%$$

where N_0 and N are the number of colonies in the control and experimental groups, respectively. Each colony analysis test was repeated three times.

MIC and MBC determination

MIC and MBC were determined by a standard broth microdilution assay in 96 well plates. Log phase bacteria were prepared at 1×10^5 CFU mL^{-1} in nutrient broth. MQDs were dispersed in PBS, sterilized by 0.22 μm filtration, and two-fold serially diluted to give final concentrations of 320 to 0.625 μg mL^{-1} after inoculation. Bacteria only and ampicillin (4 μg mL^{-1}) were used as negative and positive controls, respectively, and medium only wells served as blanks. For the dark group, plates were incubated at 37 °C overnight. For the light group, plates were irradiated with a 450 nm LED (50 mW cm^{-2}, 40 min) and then incubated at 37 °C overnight in the dark. OD_{600} was recorded using a

microplate reader. MIC was defined as the lowest concentration giving an OD_{600} comparable to the medium blank, with no visible turbidity. For MBC, aliquots from wells at and above the MIC were plated on agar and incubated at 37 °C for 24 h. MBC was defined as the lowest concentration yielding no visible colonies.

Light on–off redox cycling assay

For reversible light on–off cycling experiments, the MQDs–TMB system was alternately irradiated with 450 nm light (0.1 W cm^{-2}, 20 min) and incubated in the dark (20 min). UV–vis absorption spectra were recorded after each step, and the procedure was repeated for three cycles.

ROS scavenging of MQDs

•DPPH scavenging. 0.05 mg/mL of •DPPH alcoholic solution was mixed with different concentrations of MQDs (1, 2, 3, 4, 5, 6, 7, 8, 9, 10, and 11 μg mL^{-1}). The residual •DPPH was spectrophotometrically monitored at 517 nm using UV-vis spectroscopy. The EC_{50} value of MQDs was calculated by the software Graphpad Prism.

•OH scavenging. •OH was generated using the classical Fenton reaction. Salicylic acid trapped the short-lived •OH and generated 2,3-dihydroxybenzoic acid with a characteristic absorption peak at ~510 nm in the UV-vis spectrum. MQDs solutions (0, 10, 20, 40, 60, and 80 μg mL^{-1}) were added with 600 μM of $FeSO_4$ and 587 μM of H_2O_2, mixed thoroughly and then reacted at room temperature for 1 h. Then 600 μM of salicylic acid was added to the solution. The residual OH can be determined by measuring the absorbance at 510 nm using UV-Vis spectroscopy.

•O_2^- scavenging. The ability of MQDs to scavenge •O_2^- was determined through WST-1 using a colorimetric SOD assay kit according to the manufacturer's instructions. •O_2^- was produced via the oxidation of xanthine with xanthine oxidase (XOD), and reacted with WST-1 to generate yellow formazan. The amount of formazan generated was directly correlated to the amount of •O_2^- produced in the reaction system. Therefore, WST-1, xanthine, and XOD were added to MQDs （0, 5, 10, 20, and 40 μg mL^{-1}). The amount of formazan could be measured at 450 nm by UV-vis spectroscopy.

H_2O_2 scavenging. The H_2O_2 scavenging ability of MQDs was determined by the Catalase Assay Kit. H_2O_2 can be catalyzed by catalase to produce a red substance (N-(4-antipyryl)-3-chloro-5-sulfonate-pbenzoquinonemonoimine) with a chromogenic substrate, which has a characteristic absorption peak at ~520 nm in the UV-vis spectrum. The amount of H_2O_2 scavenged by MQDs could be calculated

from the absorbance. We briefly adjusted the dosage of the agent in the assay kit, reducing the concentration of H_2O_2 and increasing the concentration of the chromogenic substrate. The concentration of the H_2O_2 solution was 1 mM, while the concentrations of MQDs were 0.03, 0.06, 0.12, and 0.18 mg mL^{-1}, respectively.

Biocompatibility of MQDs

In vitro cytocompatibility. The cytocompatibility of MQDs was evaluated using a Cell Counting Kit-8 (CCK-8) assay. Endothelial cells (ECs) and human keratinocyte cells (HaCaT) were seeded in 96-well plates at a density of 8×10^3 cells per well and incubated for 24 h. The culture medium was then replaced with fresh medium containing MQDs at concentrations of 0, 20, 40, 60, 80, 100, 120, and 140 μg mL^{-1}. After an additional 24 h incubation, the cells were rinsed twice with pre-warmed PBS and incubated with fresh medium containing CCK-8 working solution for 2 h. The absorbance at 450 nm was measured, and the relative cell viability was calculated. To determine the half-maximal inhibitory concentration (IC_{50}), the concentration range of MQDs was further extended up to 480 μg mL^{-1} in the CCK-8 assay. The IC_{50} value of MQDs toward HaCaT cells was determined to be 243.33 μg mL^{-1}.

In vivo biocompatibility. Female BALB/c mice (6–8 weeks old) were housed under standard conditions with free access to food and water and acclimated for 7 days prior to experiments. MQDs were administered via tail-vein injection at a dose of 5 mg kg^{-1}. At 24 h and 7 days post-injection, blood samples were collected via orbital bleeding and centrifuged at 3500 rpm for 10 min to obtain plasma. Major organs, including liver, kidney, and spleen, were harvested and stored at −80 °C. Plasma and organ samples were digested prior to analysis, and the Ti content was quantified by inductively coupled plasma mass spectrometry (ICP–MS), C (μg L^{-1}). After acid digestion, each blood or organ sample was diluted to a final volume of V_0=10 mL. The total Ti amount in each organ was calculated as:

$$m_{Ti,organ} = C \times \frac{V_0}{1000} \times f$$

Where f is the dilution factor (here $f = 1$).

The injected Ti amount was calculated from the dosing level of MQDs and the Ti mass fraction determined by ICP–OES:

$$m_{Ti,inj} = D_{MQDs} \times w_{Ti} \times BW$$

Where D_{MQDs} = 5 mg kg^{-1}, w_{Ti} = 0.2385, and BW is the mouse body weight. The percentage of injected dose in each organ was then calculated as:

$$\%ID = 100 \times \frac{m_{Ti,organ}}{m_{Ti,inj}}$$

Hemolysis assay

The erythrocytes were collected by centrifugation at 2000 rpm for 10 minutes and then washed with PBS until the supernatant became transparent. Dilute the erythrocytes with PBS to obtain a 2% (v/v) working solution. Then different concentrations of MQDs were mixed with 200 µL of erythrocytes suspension in a 96-well plate and incubated at 37°C for 2 hours. After incubation, the mixture was centrifuged at 2000 rpm for 15 minutes. After incubation, the mixture was centrifuged at 2000 rpm for 15 minutes. 100 µL of supernatant was collected into a new 96-well plate and absorbance was measured at 545 nm.

Erythrocytes treated with 2% Triton-X 100 and PBS served as positive and negative controls, respectively. The experiment was repeated three times for each group. The hemolysis ratio of erythrocytes was calculated using the following formula:

$$\text{Hemolysis assay (\%)} = \frac{A_s - A_n}{A_p - A_n} \times 100\%$$

where A_n, A_p, and A_s are the optical density values of erythrocytes treated with PBS, Titron X-100, and sample (MQDs), respectively.

Anti-inflammatory capacity

Quantitative real-time PCR (qRT-PCR). Total RNA from human macrophages was extracted using TRIzol (Thermo Fisher Scientific, USA) reagent according to the manufacturer's protocol. Total RNA was reverse-transcribed into cDNA using the RevertAid First Strand cDNA Synthesis Kit (Thermo Fisher Scientific, USA). The primer sequences used are listed in Table 1. The qRT-PCR reaction was carried out using SYBR Green Premix *Pro Taq* HS qPCR Kit (Accurate Biology, China). Each qRT-PCR reaction was performed in triplicate as follows. Step 1: denaturation at 95°C for 10 min, step 2: 40 cycles of 95°C for 15 s and 60°C for 1 min. GADPH was used as an endogenous control/ internal reference. The relative expression level was computed using the 2-ΔΔCt method.

Table 1 Gene primers sequence qRT-PCR analysis.

Gene primer	primer sequence FP（5’-3’）	primer sequence RP（5’-3’）
IL-8	CAGTTTTGCCAAGGAGTGCTAA	AAACTTCTCCACAACCCTCTGC
IL-6	GCCACTCACCTCTTCAGAACGA	TCACCAGGCAAGTCTCCTCATT
IL-1β	TACCTGTCCTGCGTGTTGAAA	GGTGCTGATGTACCAGTTGGG
TNF-α	GCTGCACTTTGGAGTGATCG	ATGAGGTACAGGCCCTCTGA
GAPDH	GGAAGCTTGTCATCAATGGAAATC	TGATGACCCTTTTGGCTCCC

Flow cytometry analysis. J774a.1 cells incubated with MN (the concentrations of MQDs were 0, 40, and 80 μg mL^{-1}) were stimulated with Lipopolysaccharide (LPS, Sigma-Aldrich, USA) 1μg $mL^{-1}$for 24 hours. After collection of the cells, they were stained using M1 marker antibody CD86 (AB_10563077, BD Pharmingen™, USA) and M2 marker antibody CD206 (AB_398476, BD Pharmingen™, USA) for 10 min. Then, the Fluorescence intensity of PE-CY™7 (561128, BD Pharmingen™, USA)and APC (550889, BD Pharmingen™, USA)dyes were detected by a flow cytometer.

Preparation of MQDs@Col-SA MN

In order to load MQDs in a polymer network to form a needle tip that could reach the wound tissue directly, a two-step casting process was carried out. At first, a mixture of MQDs and Col/SA solution was filled into the polydimethylsiloxane (PDMS) mold to form the tips. Secondly, the mixture of PVA and PVP was applied to the mold to form the pedestals. The MN was composed of rectangular pyramid needles with a height of 600 μm.

In vivo assessment of diabetic wound healing.

The female nonobese diabetic (NOD) and Institute of Cancer Research (ICR) mice were purchased from Shanghai Slack Laboratory Animal Co., Ltd. [SCXK (Shanghai) 2022 - 0004]. The mice were housed and cared for at the Experimental Animal Centre of Zhejiang University of Traditional Chinese Medicine [license number: SYXK (Zhejiang) 2021-0012].

The ability of the microneedles to accelerate chronic wound healing was assessed by establishing a full-thickness skin wound model in spontaneously diabetic mice. All animal procedures were

administered and discarded according to the guidelines of the national animal research code and approved by Zhejiang Chinese Medical University Laboratory Animal Research Center (approval number: IACUC-20241118-28). At first, after adaptive feeding for one week, those mice were fed with high-fat chow and housed in the animal facility. The blood glucose levels of mice were continuously measured until their glucose level was>16.7 mM, which suggested the successful establishment of diabetes. The mice were then randomly divided into four groups: negative control group, NOD operation group, standard wound treatment group (gauze cloth with Ethacridine Lactate (EL)), and MQDs treatment group (3 mice per group).

Skin injury model assessment. After 3, 7, 10, and 14 days, digital images of wounds were acquired and the wound area was calculated using ImageJ. The percentage of wound size was calculated as follows: $[A_{(3,7,10,14)}/A_0] \times 100\%$, where A_0 and $A_{(3,7,10,14)}$ represent the wound areas on day 0 and days 3, 7, 10, and 14, respectively.

HE and Masson staining. The wound tissues after 14 days of treatment were stained with Hematoxylin-Eosin (HE) for histological examination following established procedures. Then, the stained sections were used to capture tissue images with a Leica microscope. Masson's trichromatic stain was utilized to evaluate collagen deposition activities in wound healing.

qRT-PCR. Total RNA from mouse skin tissues was extracted using TRIzol reagent according to the manufacturer's protocol. Total RNA was reverse-transcribed into cDNA using the RevertAid First Strand cDNA Synthesis Kit. The primer sequences used are listed in Table 2. The qRT-PCR reaction was carried out using SYBR Green Premix *Pro Taq* HS qPCR Kit (Accurate Biology, China). Each qRT-PCR reaction was performed in triplicate as follows. Step 1: denaturation at 95°C for 10 min, step 2: 40 cycles of 95°C for 15 s and 60°C for 1 min. GADPH was used as an endogenous control/ internal reference. The relative expression level was computed using the 2-ΔΔCt method.

Table 2 Gene primers sequence qRT-PCR analysis.

Gene primer	primer sequence FP (5'-3')	primer sequence RP (5'-3')
IFN-γ	GGAGGAACTGGCAAAAGGATGC	TGTTGCTGATGGCCTGATTGTC
IL-6	GAGACTTCCATCCAGTTGCCTTC	TGTTGGGAGTGGTATCCTCTGTG

IL-1β	TCGCAGCAGCACATCAACAAG	TCCACGGGAAAGACACAGGTAG
TNF-α	ATCCGCGACGTGGAACTG	ACCGCCTGGAGTTCTGGAA
GAPDH	TGTGTCCGTCGTGGATCTGA	TTGCTGTTGAAGTCGCAGGAG

Western Blot. The expressions of interleukin-1β (IL-1β), p65, p-p65, interleukin-6 (IL-6), tumor necrosis factor-α (TNF-α) and Nuclear factor erythroid-derived 2-like 2 (NRF2) were detected by western blot. After tissue homogenization and centrifugation, the supernatant was collected for total protein. After treatment, total protein extracts were obtained using the BCA protein assay kit (P0010, Beyotime, China), and the concentration of isolated proteins was measured. Then, 50 μg of protein from each sample was loaded onto an SDS-PAGE gel for electrophoresis. The separated protein was transferred to a PVDF (Immun-Blot®, Bio-Rad, USA) membrane. After blocking the nonspecific binding site with a protein-free rapid blocking buffer (Servicebio®, China), the membrane was incubated with primary antibody (NF-κB p65 (CST, 1:1000, USA), Phospho-NF-κB p65 (Ser529, CST, 1:1000, USA), TNF-α (Proteintech, 1:1000, China), IL-1β (Beyotime, 1:1000, China), IL-6 (Beyotime, 1:1000, China), NRF2(CST, 1:1000, USA), HSP90 (protein tech, 1:2000, China), β-actin (Cohesion, 1:2000, England)) at 4°C overnight. The membrane underwent three washes with configured tris-buffered saline with Tween 20. Subsequently, the membrane was incubated with a secondary antibody (Cohesion, 1:5000, England) coupled with horseradish peroxidase for 1 h at room temperature and developed with an ECL kit (BL523B, Biosharp, China). Ultimately, the bands were semiquantitatively analyzed by ImageJ software.

In vivo biosafety evaluation after treatment

After treatment with MQDs@Col-SA MN for 14 days, blood samples were collected for serum biochemical analysis. Liver and kidney function markers, including alanine aminotransferase (ALT), aspartate aminotransferase (AST), blood urea nitrogen (BUN), and creatinine (CRE), were measured using standard assay kits. Major organs, including liver, kidney, heart, and brain, were harvested for histological examination. Tissue samples were fixed, sectioned, and subjected to H&E staining according to standard protocols.

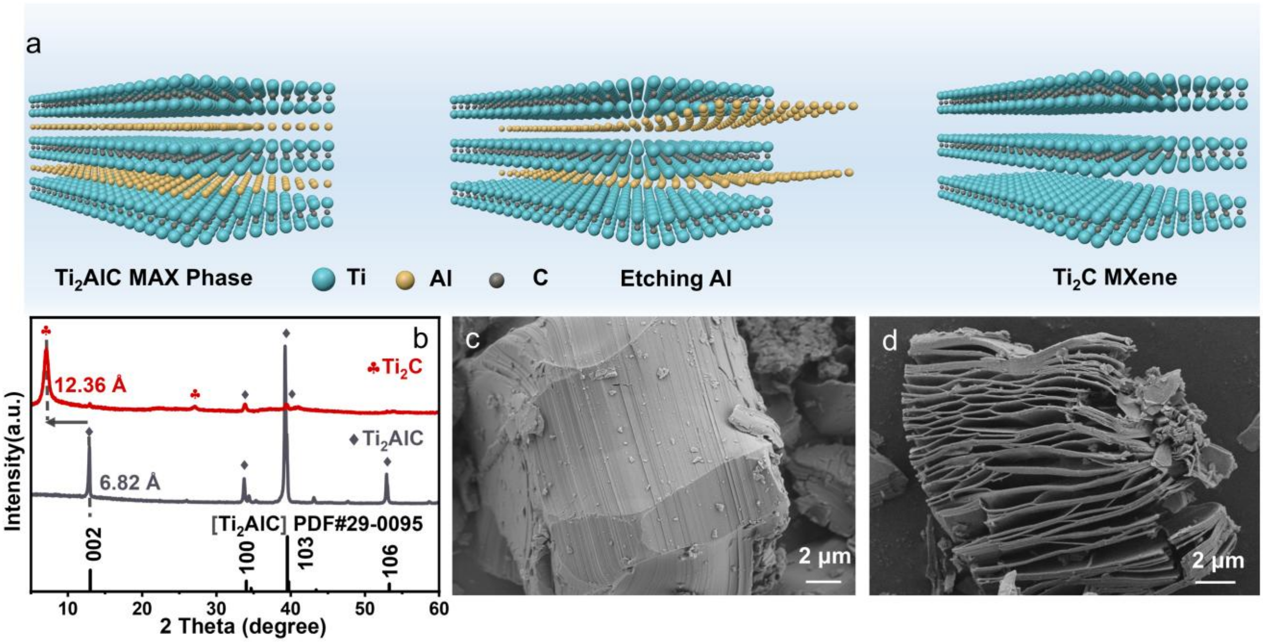

Fig. S1 a Schematic diagram of selective etching process and interlayer expansion route to prepare Ti_2C MXene from the parent Ti_2AlC MAX phase. **b** XRD diffraction patterns of Ti_2AlC MAX phase and Ti_2C MXene. SEM images of Ti_2AlC **c** before and **d** after etching.

Ti_2C MXene is synthesized by selective etching of the Al layers from the Ti_2AlC phases at room temperature (Fig. S1 a). As shown in the x-ray diffraction (XRD) patterns, the characteristic peak of the Ti_2AlC phase almost disappeared. Furthermore, the (002) peaks broadened and shifted toward lower Bragg angles compared with Ti_2AlC precursor (Fig. S1 b), which indicates an expansion of interlayers [2]. As visualized by scanning electron microscope (SEM) images, Ti_2AlC exhibits closely compacted layered platelet morphology (Fig. S1 c). After acid etching, the dense layers are converted to a loosely packed accordion-like structure (Fig. S1d). Energy dispersive spectroscopy (EDS) further corroborates the almost absence of Al in the final product (Fig. S2). All the above results confirmed the transformation from Ti_2AlC to Ti_2C.

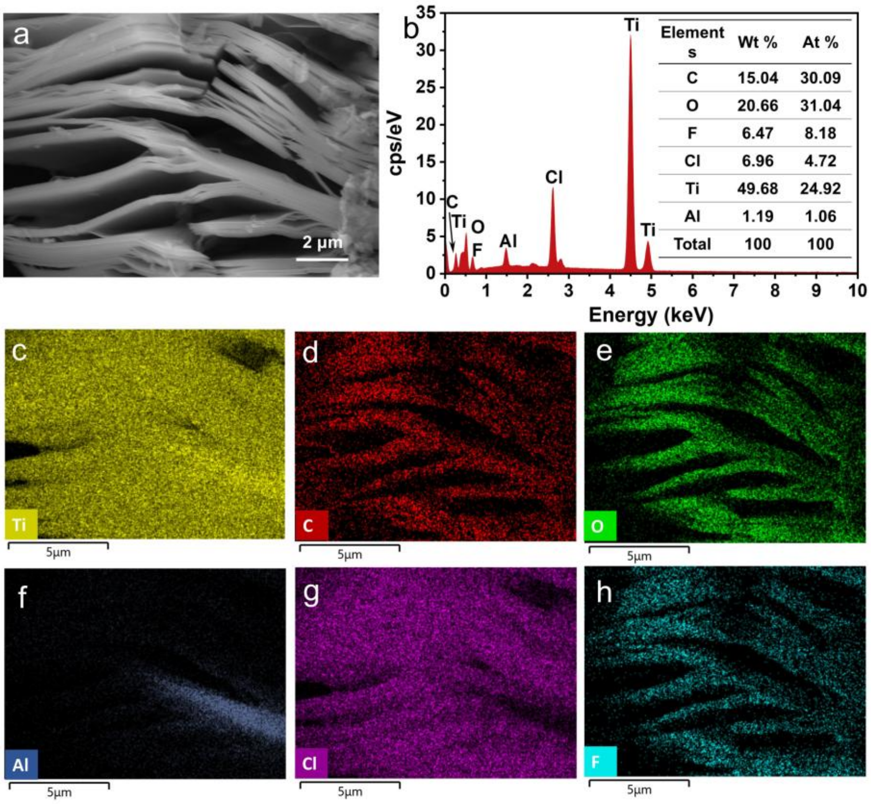

Fig. S2 a High-magnification SEM image and **b** EDS spectroscopy of Ti_2C MXene. EDS elemental mapping of **c** Ti, **d** C, **e** O, **f** Al **g** Cl and **h** F.

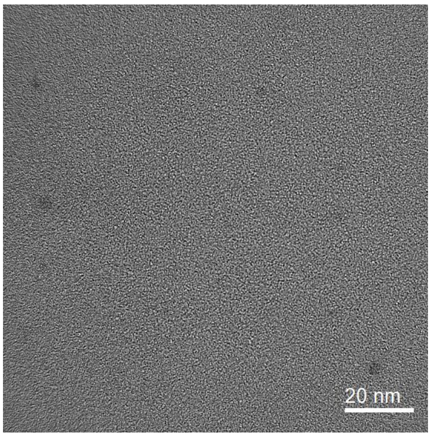

Fig. S3 TEM images of products synthesized without sodium ascorbate.

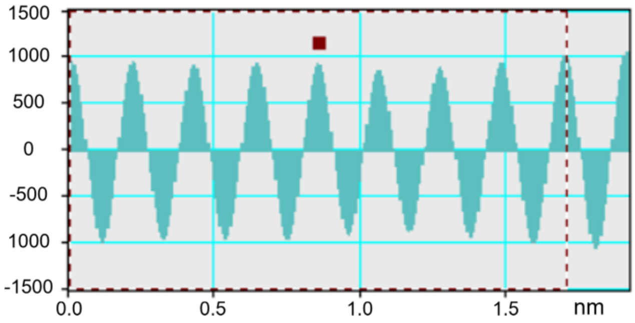

Fig. S4 Lattice spacing profiles of MQDs.

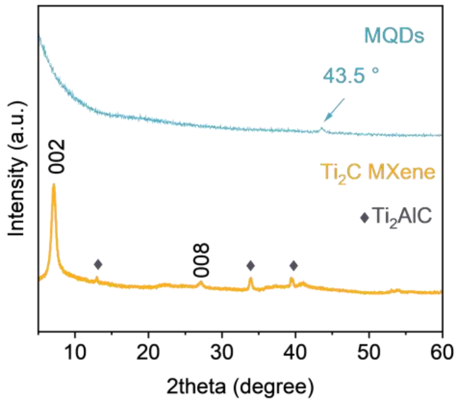

Fig. S5 XRD pattern of MQDs.

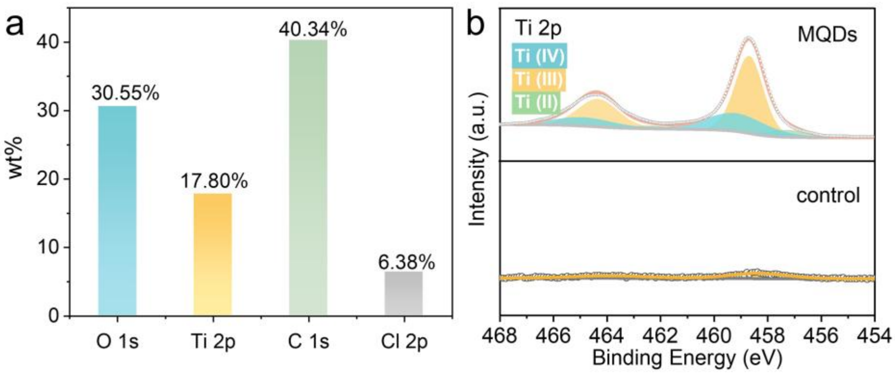

Fig. S6 a The *wt*% of each element. **b** High-resolution XPS spectra of Ti 2p region.

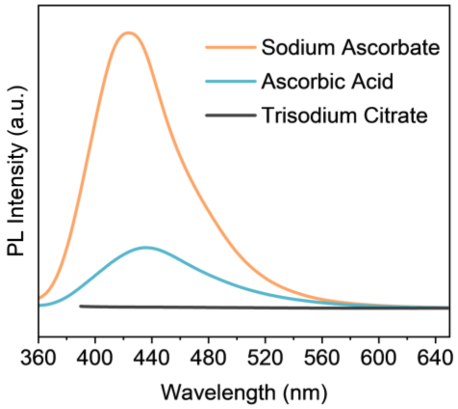

Fig. S7 Fluorescence intensity of MQDs under the protection of different additives.

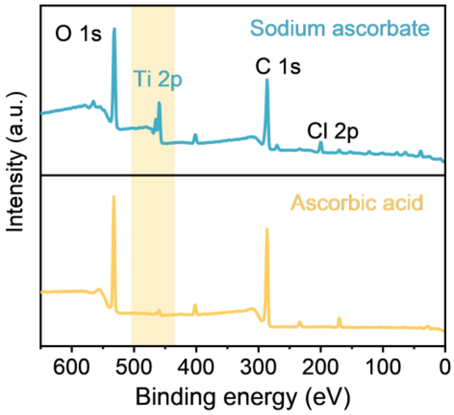

Fig. S8 Survey XPS spectra of MQDs under the protection of and Sodium ascorbate or ascorbic acid.

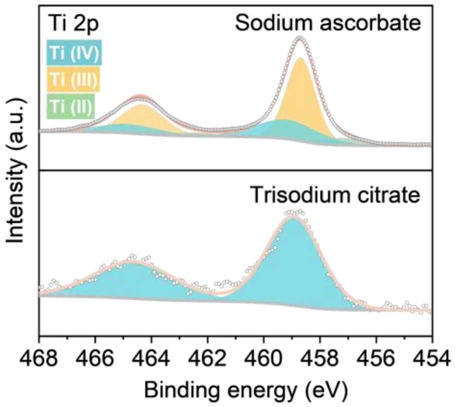

Fig. S9 High-resolution XPS spectra of Ti 2p region of MQDs under the protection of and Sodium ascorbate or Trisodium citrate

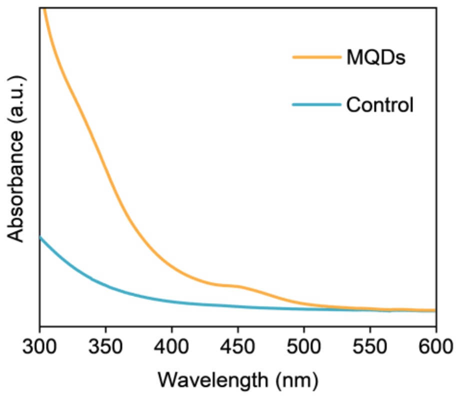

Fig. S10 UV–vis absorption spectra of MQDs and the control group.

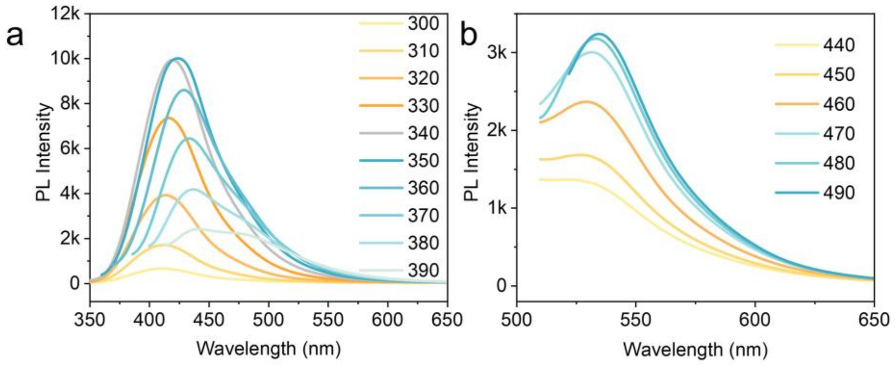

Fig. S11 a Blue and **b** green emission spectra of MQDs.

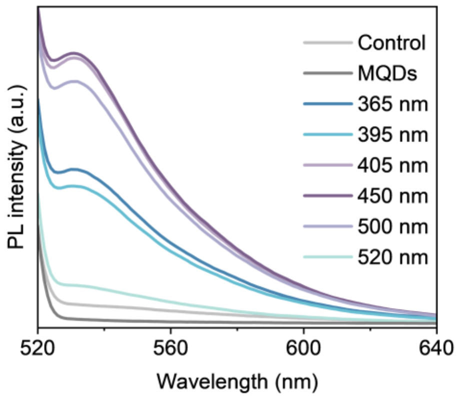

Fig. S12 $^1O_2$ generation of MQDs under different excitation wavelengths (20 mW cm^{-2}, 20 min).

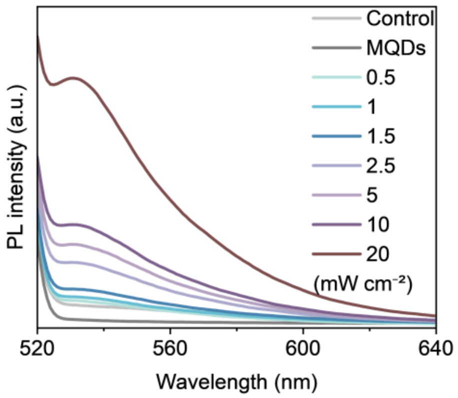

Fig. S13 $^1O_2$ generation of MQDs under 20 min irradiation at light intensities ranging from 0.5 to 20 mW cm^{-2}.

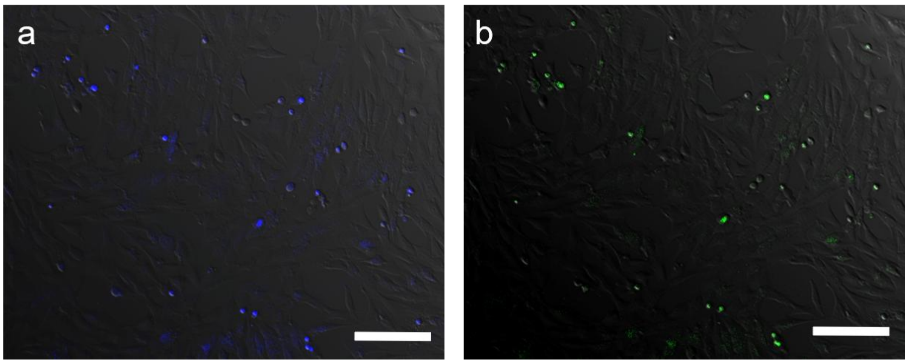

Fig. S14 a MQDs emit blue (λ_{ex}=350 nm) and **b** green (λ_{ex}=450 nm) light when endocytosed into cells. The scale bar is 250 μm.

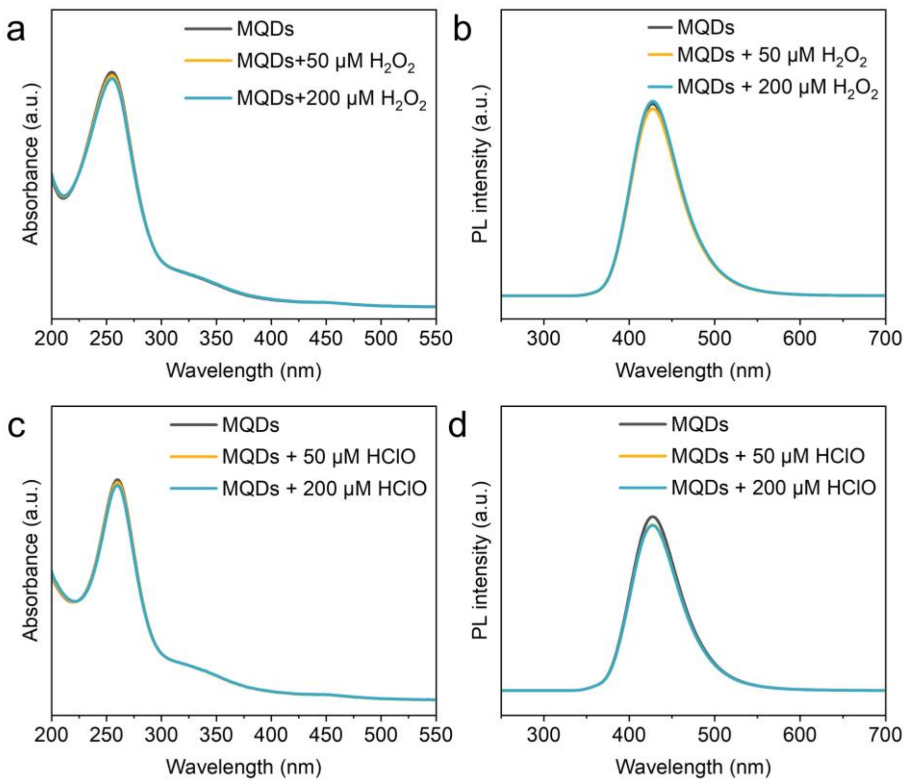

Fig. S15 a UV–vis spectra of MQDs before and after treatment with H_2O_2 (50 and 200 μM). **b** PL spectra of MQDs under the same H_2O_2 conditions. **c** UV–vis spectra of MQDs before and after treatment with HOCl (50 and 100 μM). **d** PL spectra of MQDs under the same HOCl conditions.

UV–vis and PL data show unchanged absorption-edge positions and only slight fluctuations in emission intensity after exposure to 50/200 μM H_2O_2 or HOCl (Fig. S15), demonstrating that the surface chemistry and electronic structure remain intact. As a result, the band-edge positions required for visible-light excitation are maintained, and the light-driven ROS-generation activity is not diminished.

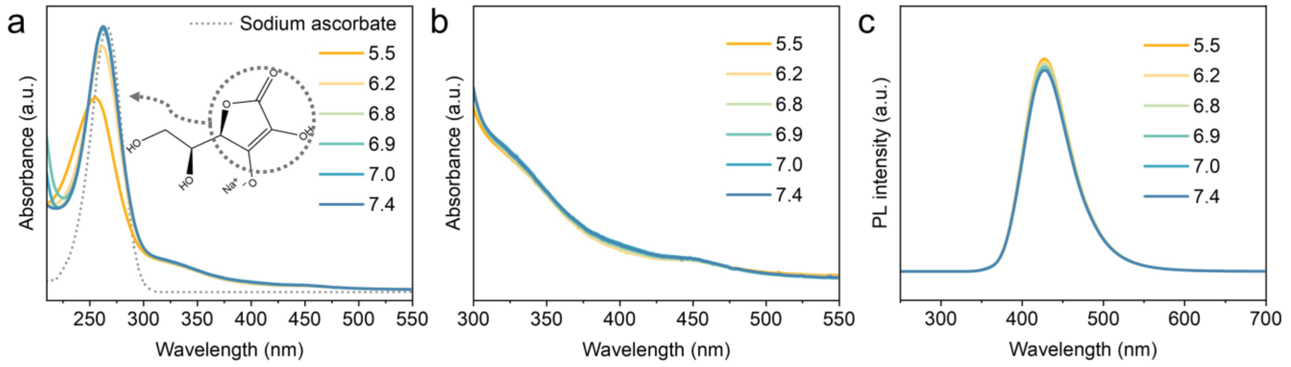

Fig. S16 a UV–vis spectra of MQDs at different pH values (5.5–7.4). **b** Enlarged view of the absorption-edge region (300-550 nm). **c** PL spectra of MQDs across the same pH range.

UV–vis absorption measurements at pH 5.5, 6.2, 6.8, 6.9, 7.0, and 7.4 reveal that the 250–300 nm absorption associated with protonation of the ascorbate-derived ligands shifts as expected [3], whereas the intrinsic 300–500 nm absorption of the MQDs remains unchanged. This confirms that the photoexcitation-relevant electronic structure is preserved across the physiological pH range, and the corresponding PL variations are minimal (Fig. S16).

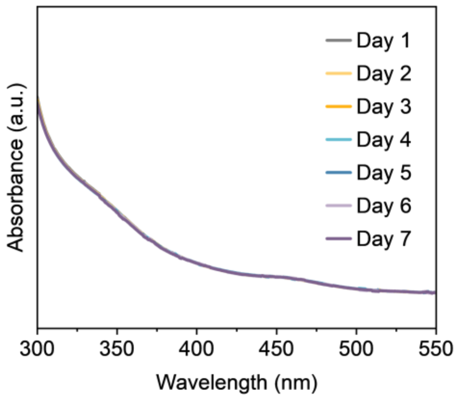

Fig. S17 UV–vis spectra of MQDs over 7 days.

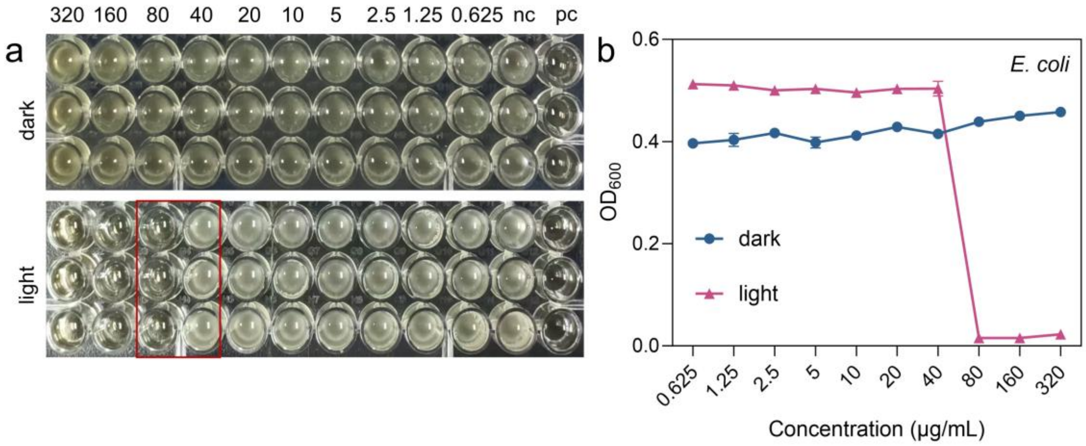

Fig. S18 a Representative 96-well plate images and **b** OD at 600 nm for MIC determination of MQDs against *E. coli* under dark and light conditions.

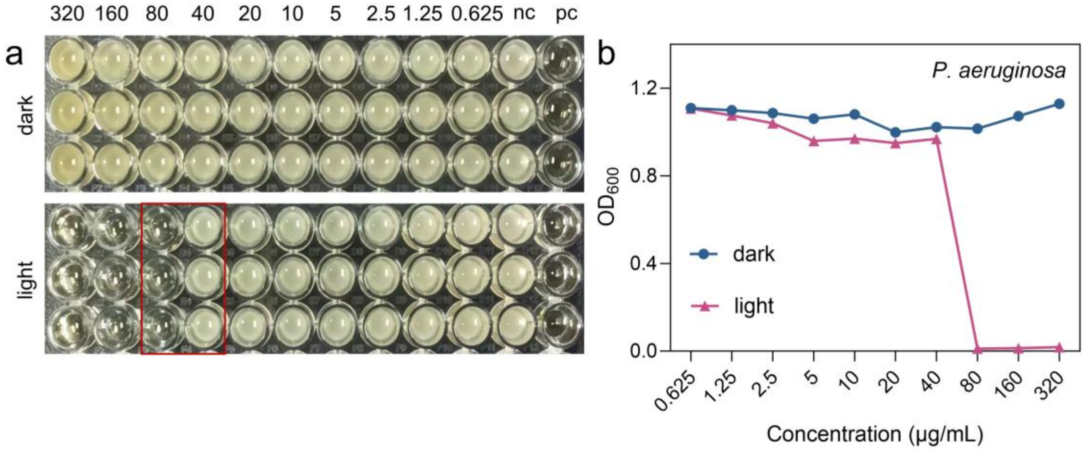

Fig. S19 a Representative 96-well plate images and **b** OD at 600 nm for MIC determination of MQDs against *P. aeruginosa* under dark and light conditions.

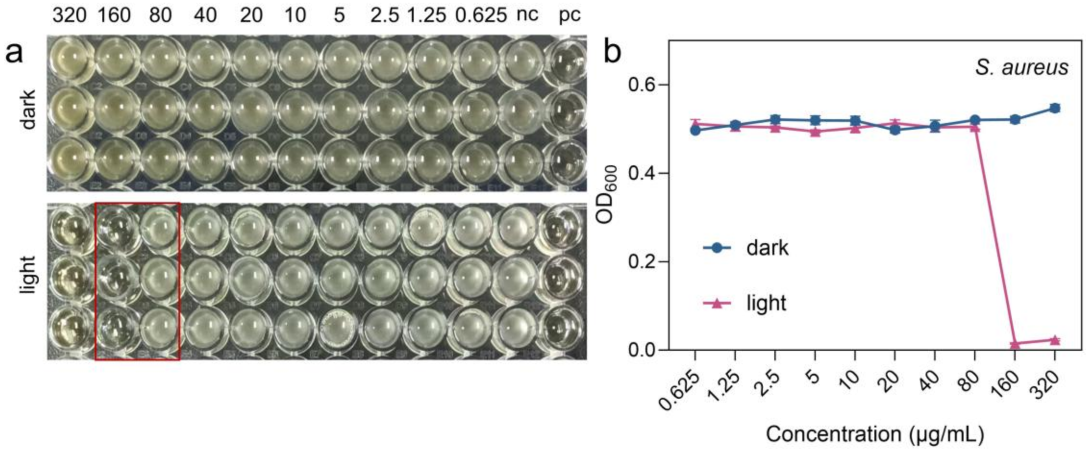

Fig. S20 a Representative 96-well plate images and **b** OD at 600 nm for MIC determination of MQDs against *S. aureus* under dark and light conditions.

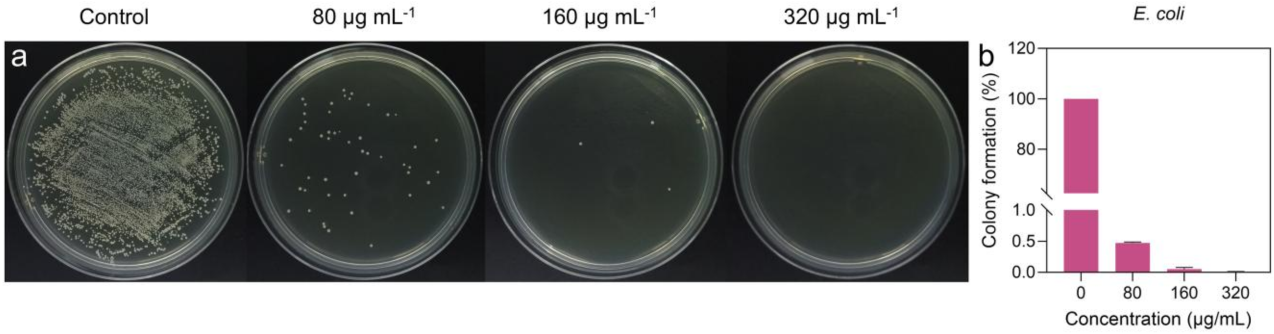

Fig. S21 a Agar plating images for MBC determination of *E. coli* after light irradiation in the presence of MQDs. **b** Corresponding colony formation at different MQDs concentrations.

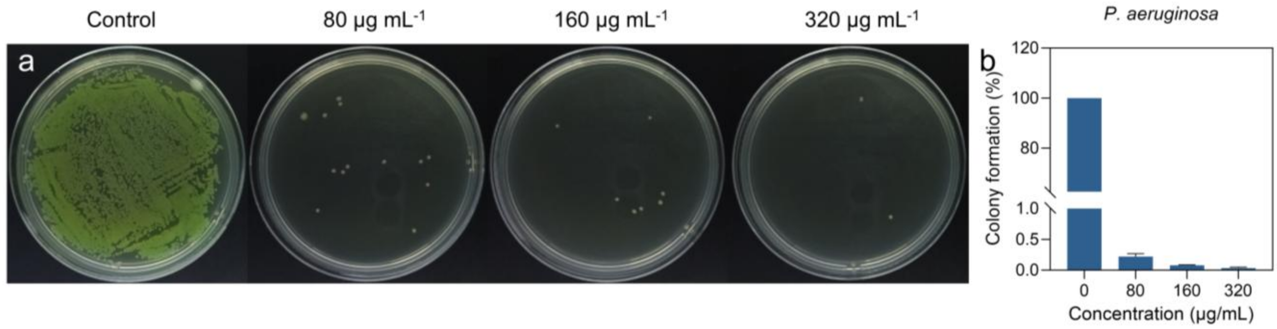

Fig. S22 a Agar plating images for MBC determination of *P. aeruginosa* after light irradiation in the presence of MQDs. **b** Corresponding colony formation at different MQDs concentrations.

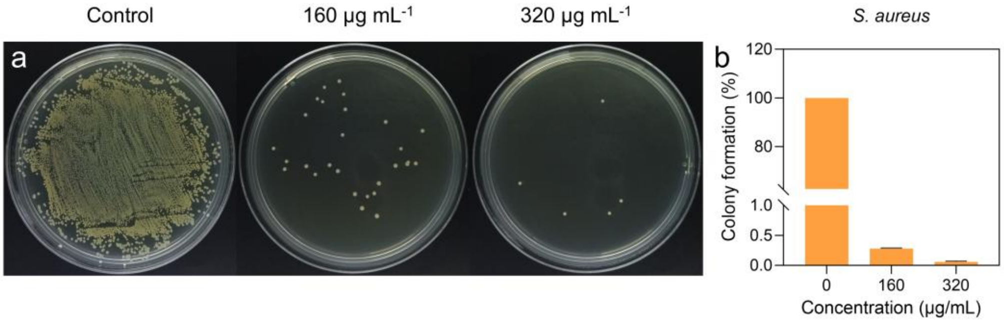

Fig. S23 a Agar plating images for MBC determination of *S. aureus* after light irradiation in the presence of MQDs. **b** Corresponding colony formation at different MQDs concentrations.

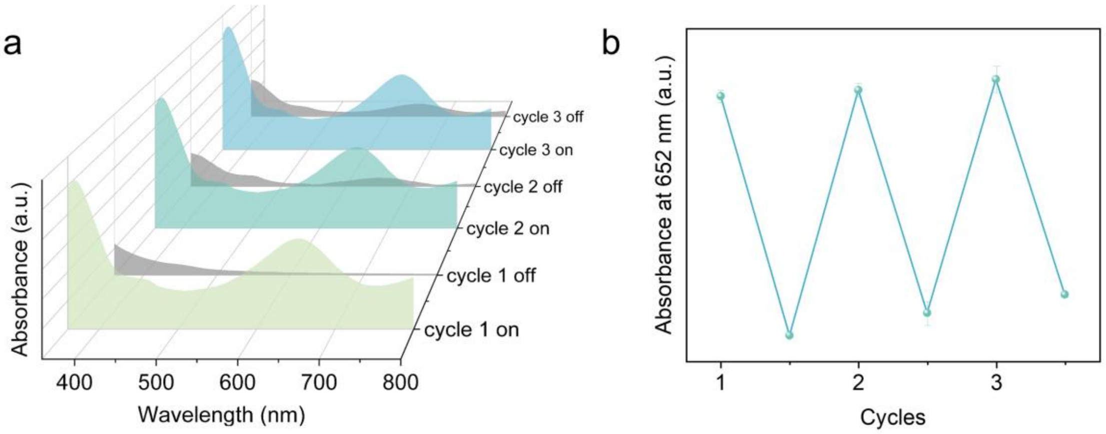

Fig. S24 Reversible photoinduced oxidation and dark reduction of TMB mediated by MQDs. **a** UV–vis absorption spectra of TMB during light on–off cycles. **b** Absorbance at 652 nm as a function of cycle number.

Under light irradiation, TMB is oxidized to oxTMB, whereas upon switching off the light, oxTMB is gradually reduced back to TMB in the presence of MQDs. This oxidation−reduction behavior can be repeatedly cycled over three consecutive light on–off sequences, without noticeable attenuation.

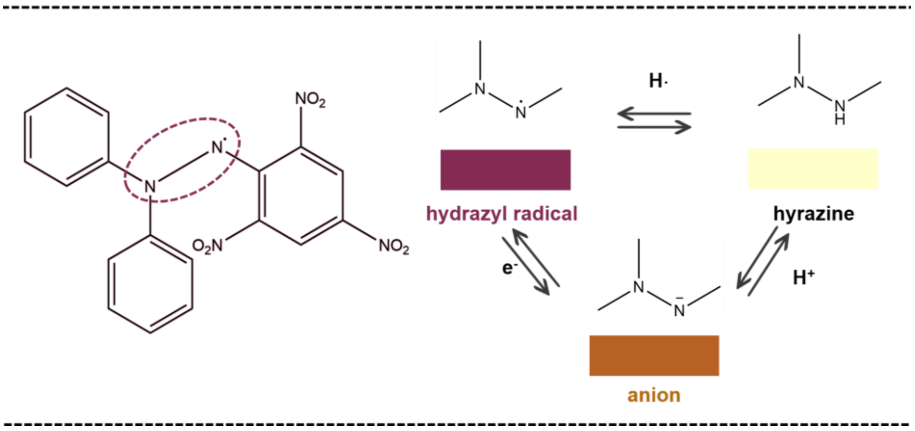

Fig. S25 Schematic diagram of ·DPPH scavenging by antioxidants.

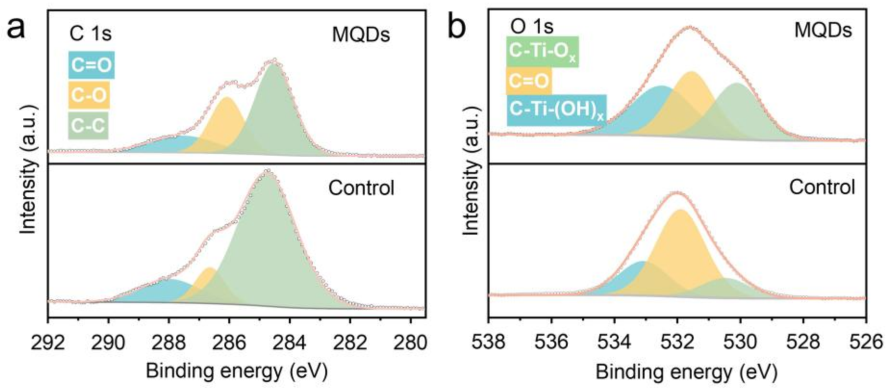

Fig. S26 High-resolution XPS spectra of **a** C 1s and **b** O 1s in MQDs and control group.

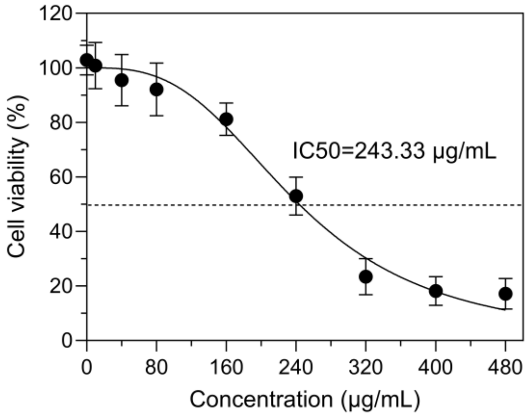

Fig. S27 IC_{50} evaluation of MQDs in HaCaT cells assessed by CCK-8 assay.

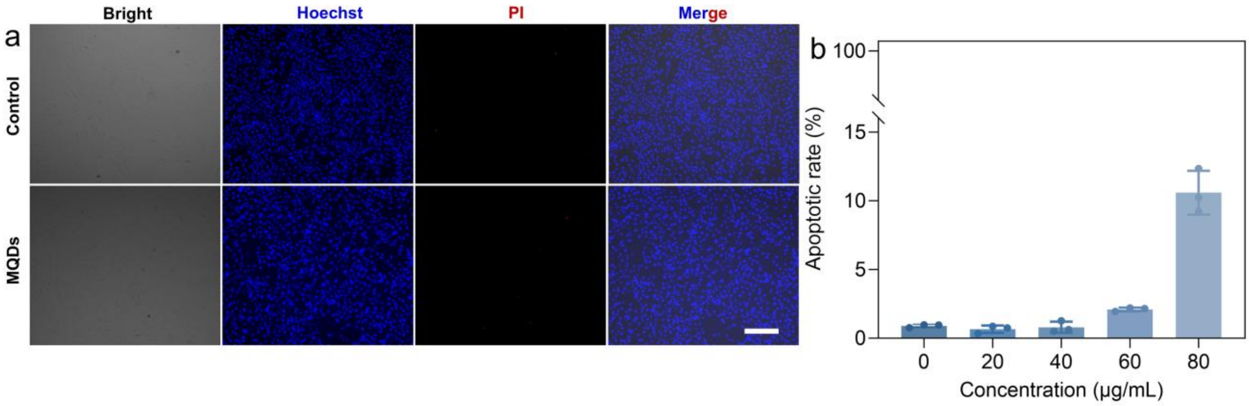

Fig. S28 a Representative CLSM images of ECs with different treatments stained with Hoechst and PI. Each experiment was repeated independently three times with similar results. **b** Apoptosis rate of ECs after incubation with different concentrations of MQDs for 24 h. (n = 3 biologically independent samples; mean ± s.d.)

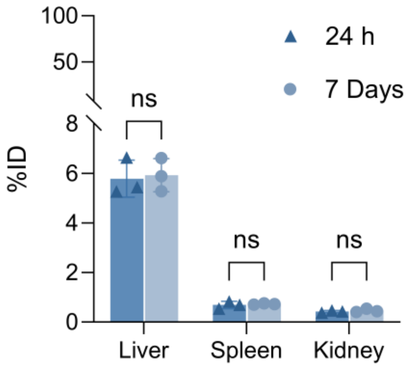

Fig. S29 Biodistribution of MQDs determined by ICP–MS at 24 h and 7 days post-administration.

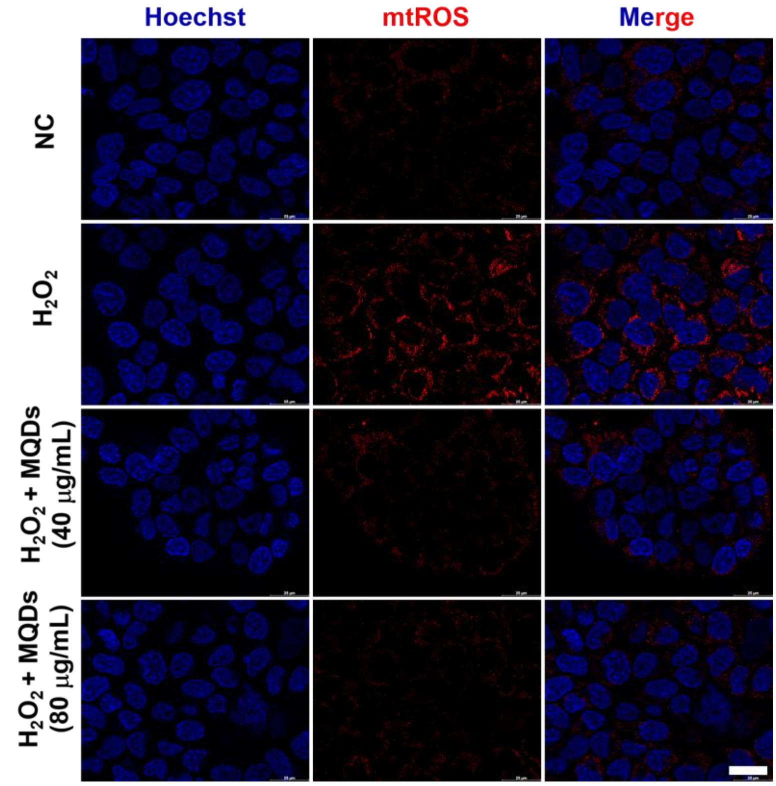

Fig. S30 CLSM images of HaCaT cells after various treatments stained with Hoechst/mtROS. Scale bar is 25 μm.

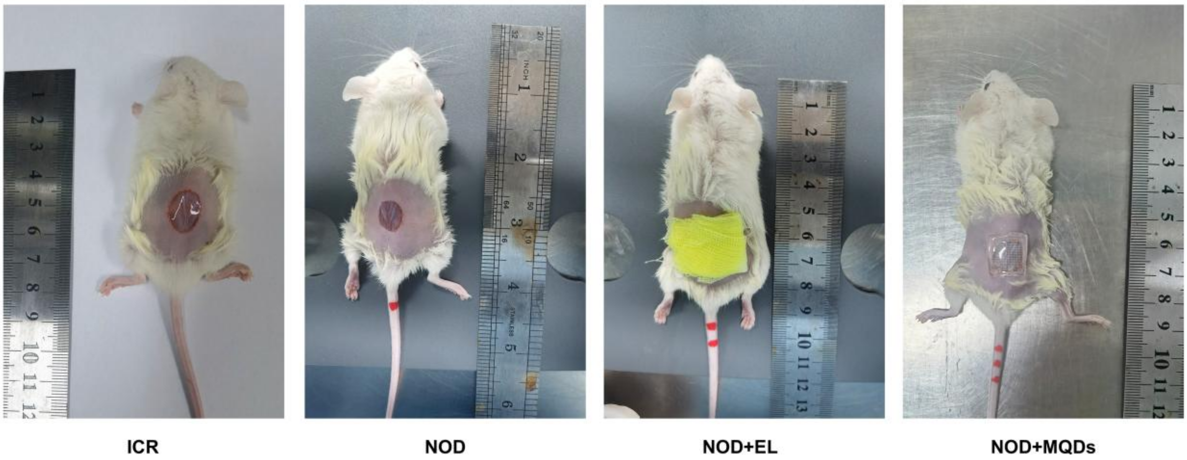

Fig. S31 Photographs of wounds in mice with different treatments.

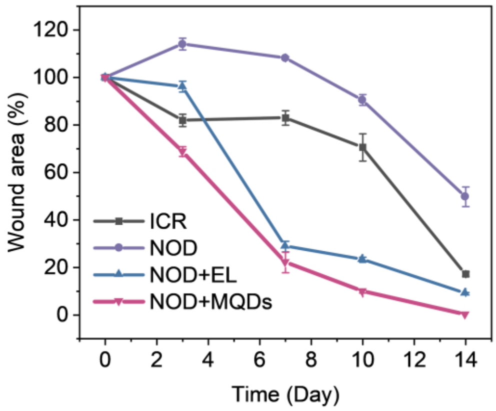

Fig. S32 Quantitative data of relative wound area of the different groups at different time points.

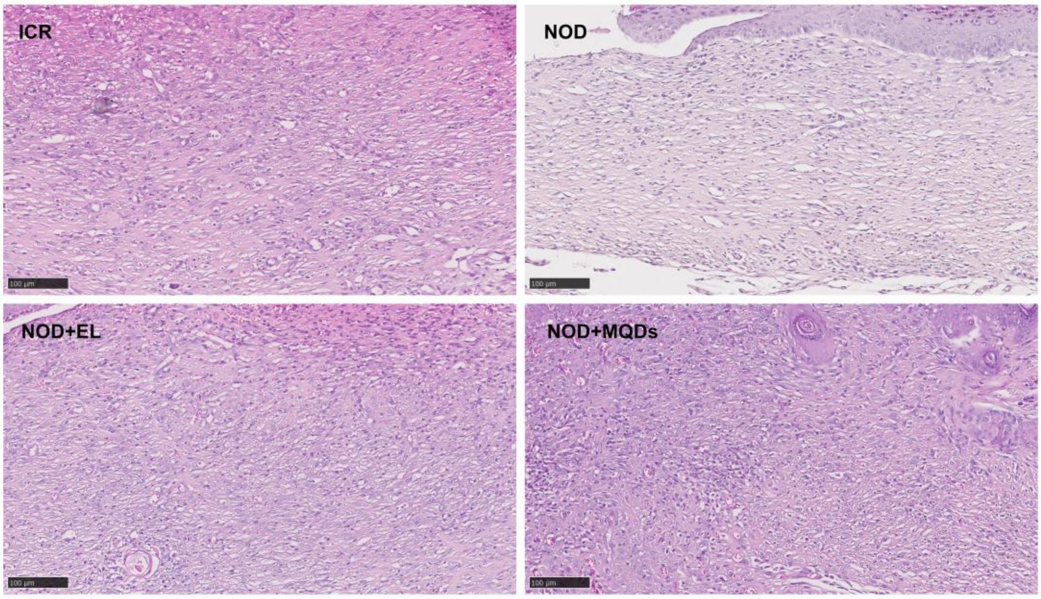

Fig. S33 Magnifications of H&E staining images of wound samples treated with different groups on the 14th day.

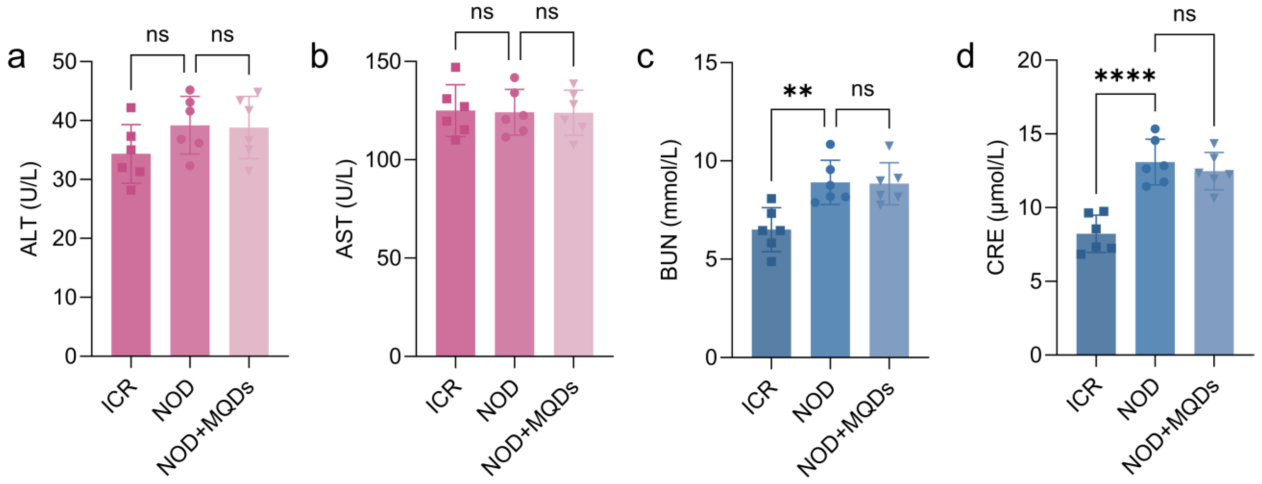

Fig. S34 Assessment of liver and kidney function based on serum **a** ALT, **b** AST, **c** BUN, and **d** CRE

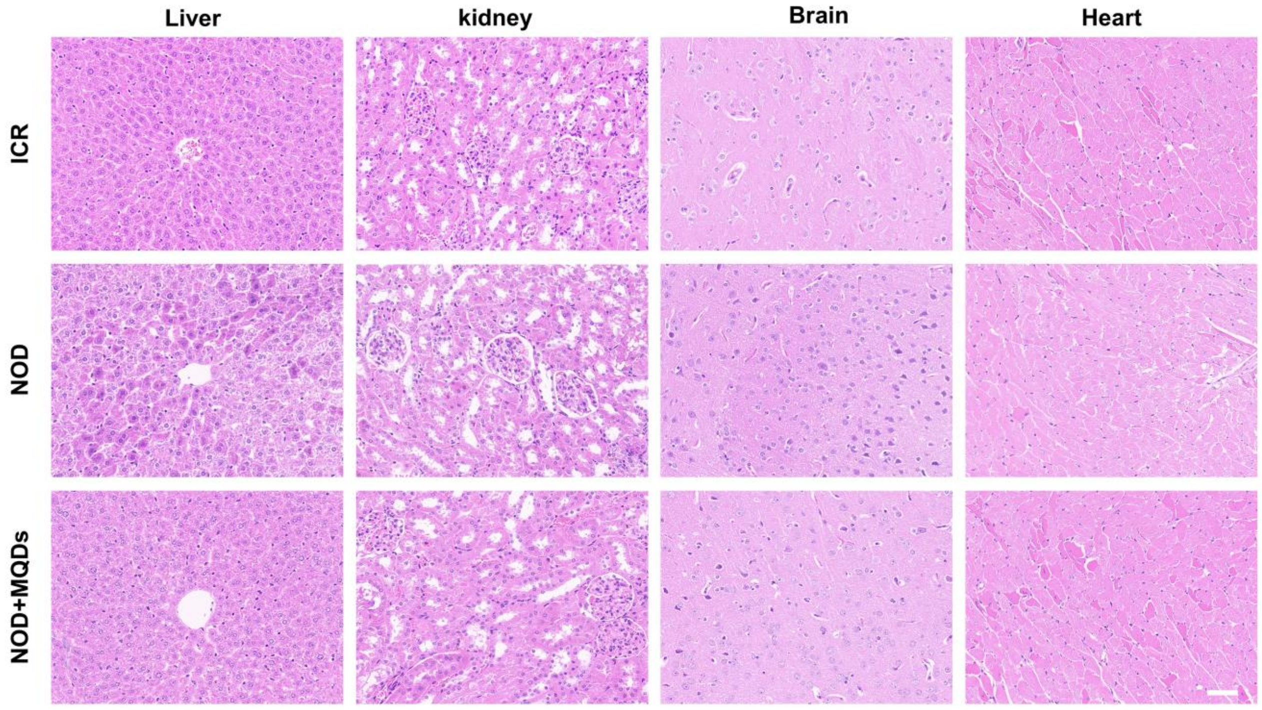

Fig. S35 Representative H&E-stained sections of the liver, kidney, brain, and heart. Scale bar is 25 μm.

Table S3. ICP–OES quantification of Ti in the MQDs dispersion.

**Parameter**	**Value**
Aliquot volume V_1	1.000 mL
Diluted volume V_0	10.00 mL
Element analyzed	Ti
ICP-OES signal C_0	1.908 mg L^{-1}
Dilution factor f	1.000
Calculated Ti concentration	19.08 mg L^{-1}
MQDs solid content	80.00 μg mL^{-1}
Ti mass fraction	23.85 wt%

Calculation:

$$C_x = C_0 \times f \times \frac{V_0}{V_1}$$

Table S4. ICP–MS quantification of Ti in plasma and major organs.

Sample	Mouse ID	Body weight (g)	Sample mass (g)	Ti in digest (µg L^{-1})	Ti content (mg kg^{-1})	%ID metric
Plasma	24 h #1	17.2	0.1895	9.2186	0.49	n.a.
	24 h #2	18.1	0.1791	8.0649	0.45	
	24 h #3	17.6	0.2249	7.7404	0.34	
	7 days #4	19.8	0.2596	4.9196	0.19	
	7 days #5	19.2	0.2536	3.2799	0.13	
	7 days #6	18.9	0.2213	5.7155	0.26	
Liver	24 h #1	17.2	0.9139	111.5730	1.22	5.44%
	24 h #2	18.1	0.8714	113.7440	1.31	5.27%
	24 h #3	17.6	0.8702	139.4634	1.60	6.64%
	7 days #4	19.8	0.8180	124.6070	1.52	5.28%
	7 days #5	19.2	0.8649	134.6000	1.56	5.88%
	7 days #6	18.9	0.8477	149.2810	1.76	6.62%
Spleen	24 h #1	17.2	0.0827	14.3660	1.74	0.70%
	24 h #2	18.1	0.0887	17.9664	2.03	0.83%
	24 h #3	17.6	0.0969	11.2927	1.17	0.54%
	7 days #4	19.8	0.0996	17.2052	1.73	0.73%
	7 days #5	19.2	0.0775	16.2597	2.10	0.71%
	7 days #6	18.9	0.0866	17.1724	1.98	0.76%
Kidney	24 h #1	17.2	0.2693	9.7330	0.36	0.47%
	24 h #2	18.1	0.2660	8.2835	0.31	0.38%
	24 h #3	17.6	0.2705	8.9302	0.33	0.43%
	7 days #4	19.8	0.2503	10.4663	0.42	0.44%
	7 days #5	19.2	0.2477	12.3172	0.50	0.54%
	7 days #6	18.9	0.2423	9.2748	0.38	0.41%

References

[1] Ibragimova R, Rinke P, Komsa H-P. Native vacancy defects in MXenes at etching conditions. Chem. Mater. 34 (2022) 2896-2906.

[2] H. Ding, Y. Li, M. Li, K. Chen, K. Liang, G. Chen, J. Lu, J. Palisaitis, P.O. Persson, P. Eklund, Chemical scissor–mediated structural editing of layered transition metal carbides. Science. 379 (2023) 1130-1135.

[3] S.A. Maniyar, J.G. Jargar, S.N. Das, S.A. Dhundasi, K.K. Das, Asian Pac. J. Trop. Biomed. 2 (2012) 220-222.
